# Supplementary material for: Modelling chemical processes in explicit solvents with machine learning potentials
Source: Nat Commun. 2024 Jul 20;15:6114. doi: 10.1038/s41467-024-50418-6 (PMC11271496; doi:10.1038/s41467-024-50418-6)
Supplement: Supplementary file 1 — Supplementary Information [file 41467_2024_50418_MOESM1_ESM.pdf]

Supporting Information

Modeling Chemical Processes in Explicit Solvents with Machine Learning Potentials

Hanwen Zhang, Veronika Juraskova and Fernanda Duarte

Contents

S1 Reactive Machine Learning Potentials (MLPs) and Solvents **2**

S2 Hyperparameters **5**

S3 Performance of Selectors **7**

S3.1 Water Models 7

S3.2 *Distance* Selector 12

S4 Benchmark Study for Diels-Alder reaction **15**

S5 The Accuracy of ACE MLPs in Implicit Solvents **17**

S6 Accuracy of ACE MLPs in Explicit Solvents **21**

S6.1 Training Strategy for ACE MLPs 21

S6.2 Reaction in Water 24

S6.3 Reaction in Methanol 30

S6.4 Summary of the explicit solvent MLPs accuracy 36

S7 Reaction Coordinate **38**

S8 2D PES **39**

S8.1 Implicit solvation 40

S8.2 Explicit solvation 41

S9 Free energy profile **43**

S10 Dynamics Studies **45**

S10.1Downhill Dynamics 45

S10.2Uphill dynamics 47

S1 Reactive Machine Learning Potentials (MLPs) and Solvents

In the last decade, MLPs have been developed to simulate various chemical processes in solution, including peptides [1, 2], spectroscopic properties [3], and chemical reactions.[4] As far as the solvent effects are concerned, most studies have employed implicit solvent, while a few have considered the solvent explicitly, either using MLPs in conjunction with QM/MM methods [3, 5] or describing the whole system with MLPs.[6, 7] Isayev and Roitberg recently reviewed the field in Ref[8]. Here, we provide a brief summary of relevant works applying MLPs to model solvent effects.

Implicit Solvent Models using MLPs:

- Noé, Clementi and colleagues [1, 2] developed an MLP-based implicit solvent model for peptides, trained on configurations extracted from explicit-solvent MD simulations. Shao *et al.* [9] applied a similar approach, using the DeepPot-SE representation to define the features of the solute structure. In addition to MM configurations, their training also included QM configurations.
- Müller and coworkers [3] introduced FieldSchNet, an MLP method capable of describing the interactions between molecules and an external field. By considering the solvent as a continuum external field with a specific dielectric constant, FieldSchNet is suitable for modelling molecules in a continuum solvent. Furthermore, the model is compatible with ML/MM approaches, as discussed below.
- Riniker *et al.* [10] introduced a graph neural network (GNN)-based implicit solvent model to simulate the dynamics of peptides. The main purpose of the model is to decrease the number of degrees of freedom compared to dynamics in explicit solvent and accelerate the sampling. The GNN model improves the base GB-Neck2 model within an Δ-learning scheme by calculating the solvation forces acting on the peptides.

Explicit Solvent with Δ-learning schemes:

- Yang *et al.* [4] utilised a Behler and Parrinello-type HDNNP to predict QM/MM potential energies for an S_N2, proton transfer of glycine, and the Claisen rearrangement reactions in explicit solvent. They first performed semiempirical QM/MM simulations, and then the free-energy profile along free-energy obtained at the SQM/MM was reweighted with NN predicted potential energies to enhance accuracy. The QM atomic charges at the SQM/MM level are introduced to NN to capture the polarisation of the QM subsystem induced by the MM environment.
- Riniker and colleagues [11] employed a Δ-learning scheme to uplift the QM energies from DFTB to various DFT methods in QM/MM simulations. The resulting potentials were validated by performing ML/MM MD simulations of retinoic acid in water and the interaction between S-adenosylmethioniate and cytosine in water.
- Corminboeuf and Ceriotti [12] trained direct and Δ-learning Behler and Parrinello-type NNPs

to reproduce energies and forces at the PBE0-D3BJ level of theory either directly, or by correcting the DFTB approach. Both models were combined within a multiple-time step algorithm to stabilize direct NNP and decrease the cost of the delta model. The resulting approach was applied to simulate the challenging properties of methanesulfonic acid in a complex mixture of phenol and H_2O_2 and investigated the role of nuclear quantum effects in hydrogen bonding. The same scheme was used to train direct and delta NNPs (GFN0-xTB to PBE0-D3BJ) to identify prevalent non-covalent interactions in a benzotelluradiazole-Cl complex in an explicit THF solvent.[13]

Direct MLP for Explicit Solvent with ML/MM Scheme:

- The FieldSchNet model introduced by Müller and coworkers [3] was used within an ML/MM approach to simulate molecular spectra accounting for solvent effects. Here, the QM region is entirely replaced by MLPs, while the solvent is considered explicitly using electronic embedding to couple the ML and MM regions.
- Meuwly and the Xie group [5] employed PhysNet ML/MM to investigate the double proton transfer within the hydrated formic acid dimer in explicit water; the solute was modelled at the MP2 level and water at the MM level. Water solvent was found to promote the first proton transfer through a favourable solvent-induced Coulomb force along the O–H$\cdots$O hydrogen bond, while the second proton transfer was significantly controlled by the O–O separation and other conformational degrees of freedom.
- Xie group [14] adopted a molecular embedding ML/MM scheme to simulate an SN2 reaction in water, further correcting the results using a weighted thermodynamic perturbation (wTP) scheme.

Direct MLP for the Whole System Including Explicit Solvents:

- Saitta *et al.* [15] proposed an ab initio protocol using DeepMD NNPs, studying the Strecker-cyanohydrin mechanism for glycine synthesis in water solution.
- We have used a kernel-based Gaussian approximation potential (GAP) to study an S_N2 reaction in solvent using a small dataset, achieving high accuracy and analysing the importance of solvent effects by comparing the difference between trajectories in implicit and explicit solvents.[16]
- Parrinello *et al.* [6] applied AIMD combined with metadynamics and several extra active learning loops to train a DeepMD for urea decomposition in explicit water.
- Yang and coworkers [7] trained a TensorMol NNP for two 1,3-dipolar cycloaddition reactions in explicit water solvent and ran MLP-MD downhill dynamics to investigate the mechanism.

Applying MLPs to chemical reaction modelling in a condensed phase is a fast-moving field of great significance for the modelling community, as the accurate description of solvent effects remains one of the grand challenges in computational chemistry. The papers mentioned above, particularly those

utilising NN-based potentials, typically require thousands of data points to obtain reliable models. In contrast, our approach, employing descriptor-based selectors combined with linear ACE, can train MLPs for the entire solvated system using only several hundred data points. This technique has the potential to advance the developments in this field, pushing the boundaries of modelling processes in solution.

S2 Hyperparameters

Table S1 presents the hyperparameter settings used in training MLPs, which are also the default values in the *mlp-train* package [17]. These hyperparameters are divided into three parts: ACE MLPs, the Smooth Overlap of Atomic Positions (SOAP) descriptor, and active learning selectors. Some of these hyperparameters have been validated in our previous works [16, 17].

For ACE MLPs, two cutoff functions are applied. The first one controls the many-body potentials and consists of an inner and outer cutoff radius. In the *mlp-train* package, the inner cutoff radius is determined based on the minimum pairwise distance observed in the training data. The outer cutoff radius r_{mb} sets the maximum distance at which the many-body potentials are considered (Table S1). The other cutoff function is applied to the pair potential, which is designed to capture long-range and short-range interactions not included in the many-body potential of the system.

The weight ratio between energy and forces in the loss function is set to 20.0. Additionally, the loss function includes L_2 regularisation with a penalty weight of 0.1. All the potentials in this study are trained using the hyperparameters listed in Table S1 unless otherwise specified.

Table S1: Hyperparameter setting for ACE potential, SOAP descriptor, and selectors. (* the polynomial degree for four- and five-body potentials do not include H)

Type	Parameter	Description	Value
ACE	ν	Maximum correlation order	4
	$D_{\nu}^{\max}$	Maximum polynomial degrees for corresponding body potential	20, 16, 16, 12 *
	r_{mb}	Outer cutoff radius for many-body potentials	4.0 Å
	r_{pair}	Cutoff radius for the pair potential	5.0 Å
SOAP descriptor	$\sigma_{\text{at}}^{\text{SOAP}}$	Spread of the Gaussian added to atomic density	1.0 Å
	$n_{\max}, l_{\max}$	The maximum number and degree for the radial (n) and angular (l) basis	6
	r_{cut}	Cutoff distance for local region	5.0 Å
energy selector	E_{T}	Selection threshold	0.1 eV
similarity selector	k_{T}	Selection threshold	0.999
distance selector	n_{neigh}	number of neighbours	15
	p_{con}	fraction of data considering as abnormal	0.2

S3 Performance of Selectors

S3.1 Water Models

In our active learning (AL) strategy for training MLPs, we employed three selectors: *energy*, *similarity*, and *distance* selectors (details in the Main text). To compare the performance of these selectors, we used the water system as an example. ACE MLPs were trained by AL using each selector with the cluster water system (see below). The validation of ACE MLPs trained by each selector consists of two parts. The first part involves assessing the accuracy of the resulting ACE MLPs for the cluster system. This assessment helps determine how accurately the ACE MLPs capture the relevant potential energy landscape. The second part examines the transferability of these ACE MLPs to the bulk water system with periodic boundary conditions (PBC). This evaluation investigates whether the ACE MLPs trained on the cluster system can accurately predict the properties and behaviour of the bulk water system. Further details about the generation of systems and the validation process for ACE MLPs will be discussed in the following sections.

Cluster system: The water cluster was generated by randomly placing 27 water molecules in a box with a size of 9.32 Å to maintain the water density. The minimum allowed distance between any two atoms in the system was set to 2 Å to avoid generating overlapping structures with large repulsive forces. Subsequently, the box size was changed to 100 Å without modifying the structure, creating a box-shaped cluster in the gas phase.

AL training was conducted using either *energy*, *similarity* or *distance* selectors (Fig. S1-S3). To assess the accuracy of the generated ACE MLPs with different selectors, ACE MLP-MD trajectory was propagated for each selector for 1 ps at 300 K. For each trained potential, energies and forces were compared to the ground truth values, computed at the PEB0-D3BJ/def2-SVP[18–20] level of theory on frames selected from ACE MLP-MD simulations every fs.

It is worth noting that the validation of the MLPs was performed for a 27-water system, for which MLP-MD trajectories were initiated from randomly placed configurations in a periodic box. This starting configuration included distorted structures, which significantly differed from the training set. In regular MD runs such structures would not be present as they are often initiated from equilibrated structures. Therefore, the corresponding errors can be considered an upper limit to the expected errors. To address concerns about the influence of using too-distorted structures as a starting point on the reported MADs, we performed additional validation on the equilibrated 27 water molecules system, using initial configurations generated by classical MD with the TIP3P water model. The errors of MLP trained by each selector are listed in Table S2, highlighting the significant improvement in MAD for equilibrated geometry compared to randomly placed structures, particularly for the similarity selector.

Bulk water: A water box was prepared by randomly placing 216 water molecules in a box size of 18.65 Å under PBC. The box was equilibrated during 20 ps of NVT simulation with a time step of 0.5 fs at 300 K using the MLP trained with the *similarity* selector. The equilibrated structure was

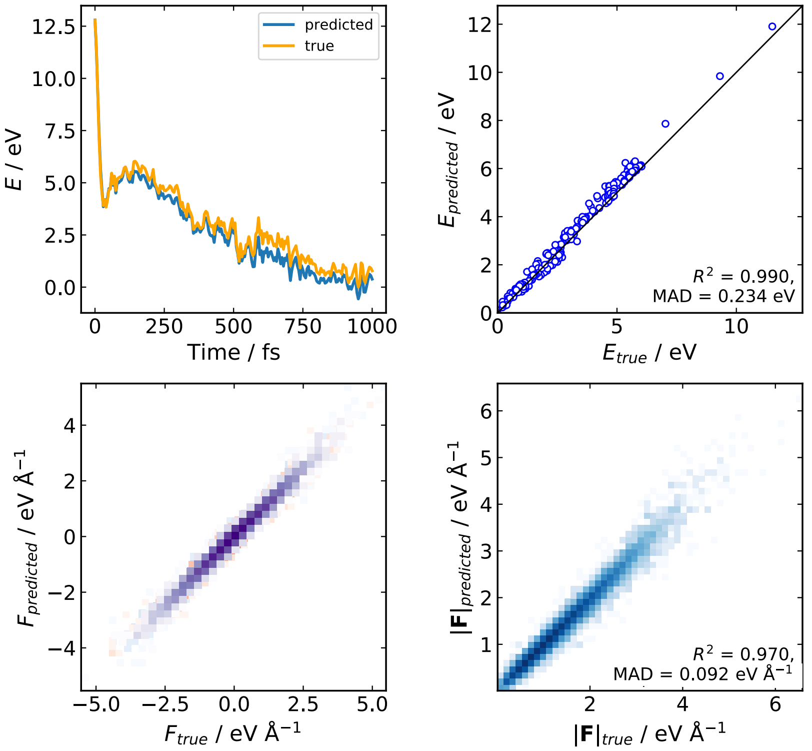

Figure S1: ACE MLP trained with active learning strategy with the *energy* selector. Comparison of ground-truth (PBE0-D3BJ/def2-SVP) and predicted (ACE MLP) energies and forces over 1 ps trajectory from a 27 random water configuration (300 K, δt = 0.5 fs).

Table S2: Errors of ACE MLPs trained with different selectors for bulk water. The initial configuration used to run the corresponding MLP-MD to generate test data included random water in a box and equilibrated water configurations.

	Random initial configuration		Equilibrated initial configuration	
	Energy MAD (meV atom^{-1})	Force MAD (meV Å^{-1})	Energy MAD (meV atom^{-1})	Force MAD (meV Å^{-1})
energy	2.89	92	2.14	85
similarity	6.31	59	1.86	81
distance	2.69	89	2.31	76

then used as a starting point to propagate three independent trajectories by ACE MLPs trained with three selectors, respectively. For each selector, MD simulations in the NVE ensemble were performed for 50 ps (stepsize 0.5 fs) to assess the stability of the trained MLP and transferability to periodic systems.

As shown in Fig. S4, the potential energy fluctuated around -1 eV to 1 eV, while the total energy remained constant with much smaller fluctuations, as expected from the NVE ensemble. These

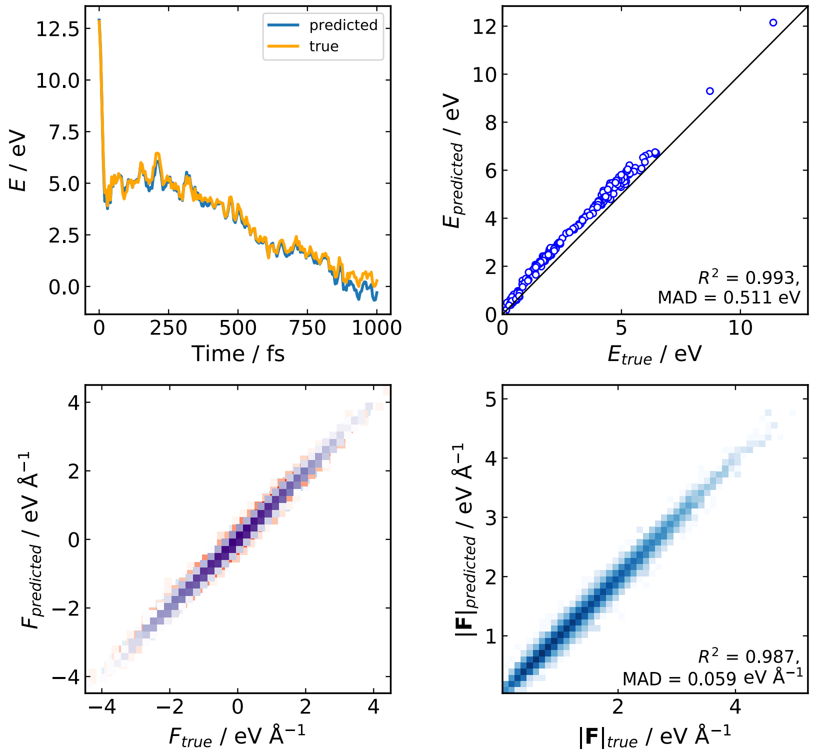

Figure S2: ACE MLP trained with active learning strategy with the *similarity* selector. Comparison of ground-truth (PBE0-D3BJ/def2-SVP) and predicted (ACE MLP) energies and forces over a 1 ps trajectory from a 27 random water configuration (300 K, δt = 0.5 fs).

results demonstrate the energy conservation in the dynamics and the stability of the potentials for much longer times than the maximum MD time used in AL loops.

In contrast to our previous study[16], where partitioning the training into intra- and intermolecular components improved MLP accuracy and stability, and accelerated the process compared to training the MLP on the entire system, we found no significant enhancement in accuracy or stability with this approach; moreover, it requires additional training time.

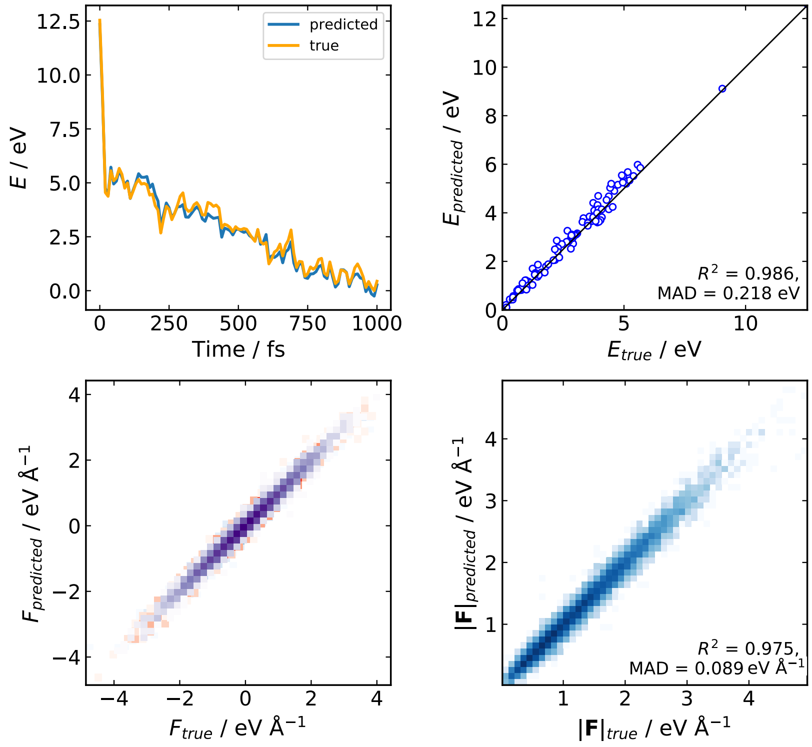

Figure S3: ACE MLP trained with active learning strategy with the *distance* selector. Comparison of ground-truth (PBE0-D3BJ/def2-SVP) and predicted (ACE MLP) energies and forces over a 1 ps trajectory from a 27 random water configuration (300 K, δt = 0.5 fs).

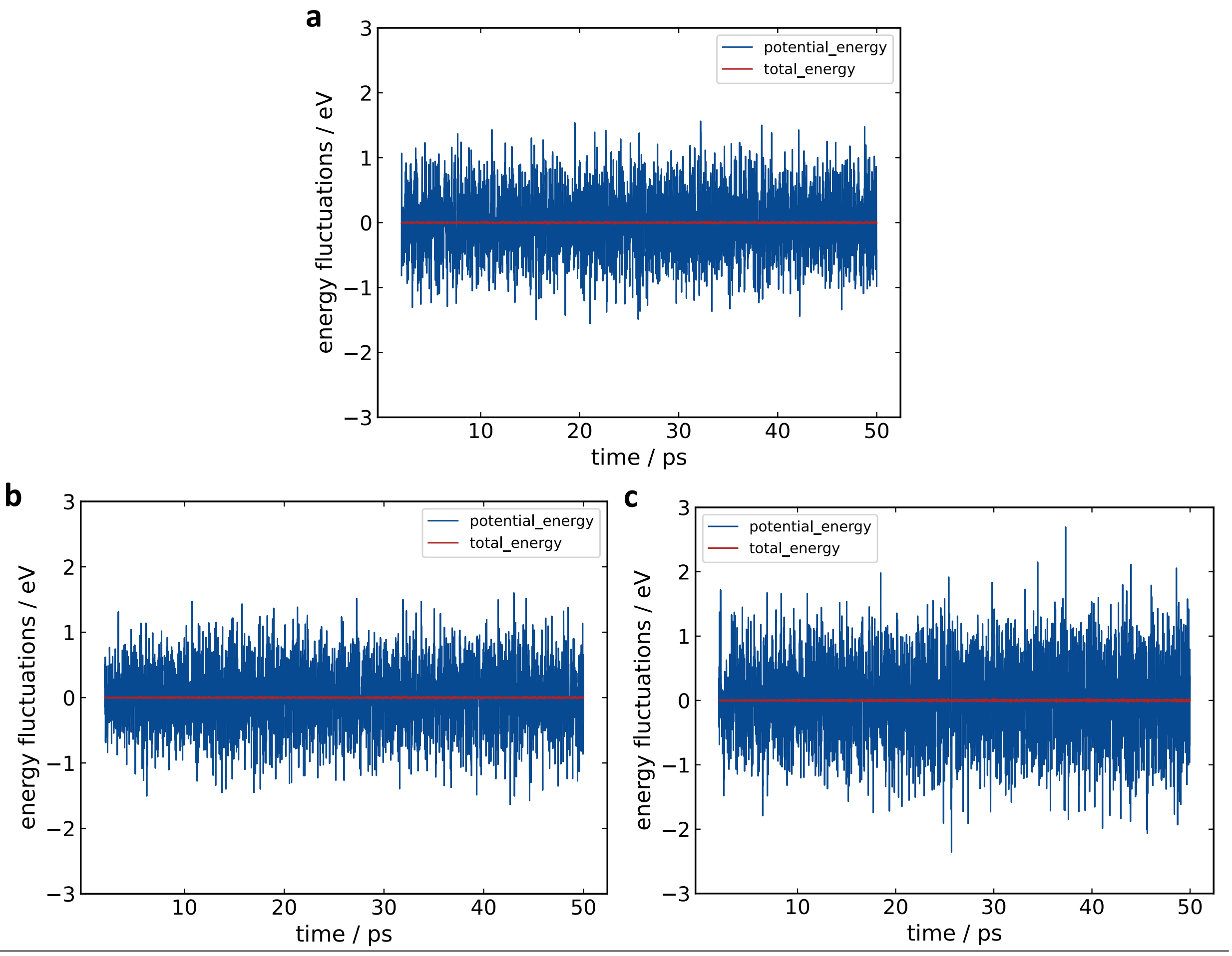

Figure S4: Evolution of Potential Energy during 50 ps of NVE simulation for a 216 water system under PBC. ACE MLP trained using (a) *energy*, (b) *similarity* and (c) *distance* selectors.

S3.2 *Distance* Selector

The distance selector presented above uses the Euclidean distance as a metric. As alternatives to the Euclidean distance, we also evaluated the performance of the cosine similarity [21] and Manhattan distance [22] (Table S3). No significant differences in data efficiency and accuracy of the resulting MLPs were observed. We attribute the similar performance to the relative simplicity of the water system and to the fact that we employed the proportion of the outliers in the dataset as a selection criterion, as opposed to a simple fixed threshold. Therefore, we chose the Euclidian metrics for the distance selector due to its simplicity and intuitive understanding of the distance in Euclidian space.

Table S3: Performance of *distance* selector using different distance metrics for a 27 water system

Distance Metrics	# Configs.	Errors	
		Energy MAD (meV atom^{-1})	Force MAD (meV Å^{-1})
Eulidean	52	2.69	89
Cosine	56	3.49	80
Manhattan	73	3.40	75

Table S4: Energy and force errors for the two versions of *distance* selectors evaluated on the same testing set generated by ACE (trained with original *distance* selector) MLP-MD for *endo* reaction of CP and MVK in gas-phase.

	Energy MAD (meV atom^{-1})	Force MAD (meV Å^{-1})
Original *distance* selector	0.55	50
Updated *distance* selector	0.36	30

Distance metrics and, consequently, *distance* selectors, may be affected by the curse of dimensionality. To address this issue, the distance selector includes an option to apply principal component analysis (PCA) for reducing the descriptor space before outlier detection. The MLPs in the gas phase reaction of CP and MVK are shown in Fig. S5 and S6 and Table S4, comparing the MLPs trained with the original *distance* selector and the updated one with PCA. Both distance selectors produce accurate MLPs. However, in cases where the system is highly complex, the updated *distance* selector is more suitable and is therefore recommended.

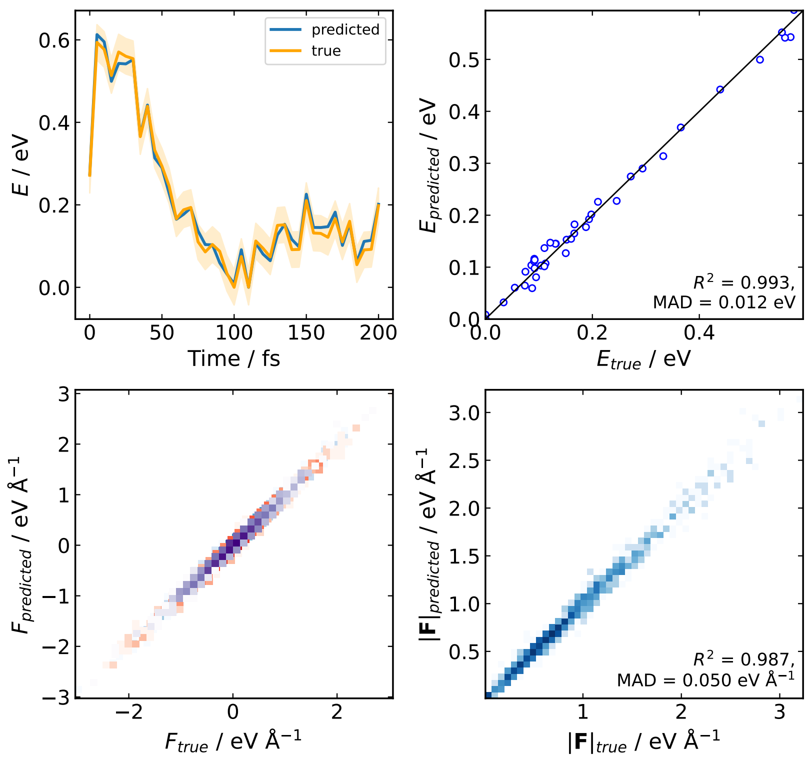

Figure S5: Performance of *distance* selector for the *endo* reaction of CP + MVK in the gas phase. Comparisons of energies and forces between ground-truth (ωB97M-D3BJ/def2-TZVP) and ACE employing original (without PCA) *distance* selector over 200-fs ACE MLP-MD trajectories started from TS (300 K, time step = 0.5 fs).

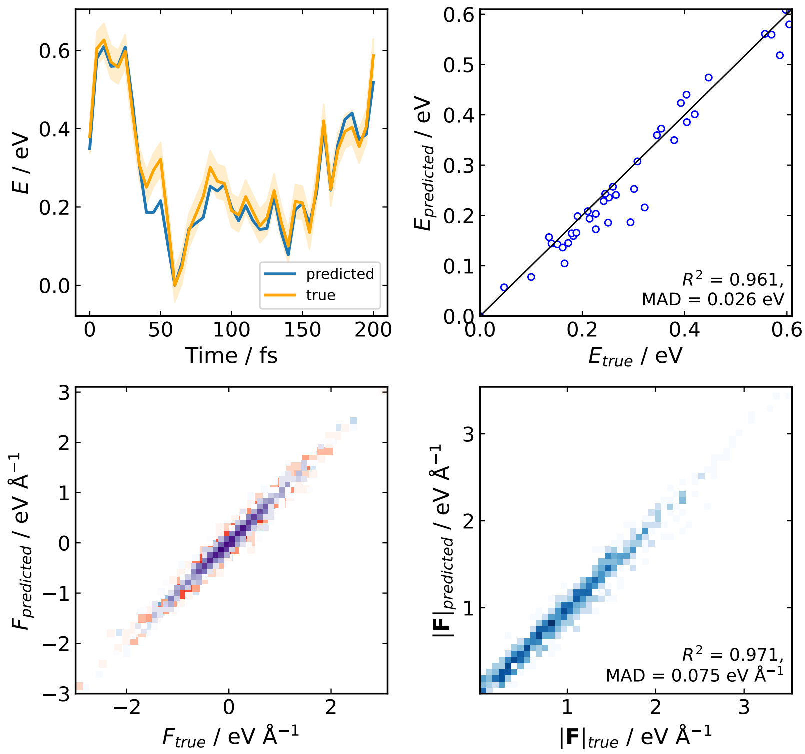

Figure S6: Performance of *distance* selector for the *endo* reaction of CP + MVK in the gas phase. Comparisons of energies and forces between ground-truth (ωB97M-D3BJ/def2-TZVP) and ACE employing updated (with PCA) *distance* selector over 200-fs ACE MLP-MD trajectories started from TS (300 K, time step = 0.5 fs).

S4 Benchmark Study for Diels-Alder reaction

To identify an efficient and accurate method for modelling the Diels-Alder (DA) reactions under study, we compared DFT performance against SCS-MP2 [23] on two properties. First, we investigated the transition state (TS) geometries obtained at different levels of theory (Table S5). Second, we compared the relative energies along the intrinsic reaction coordinate (IRC) with different levels of theory, using the configurations obtained at the PBE0-D3BJ/def2-SVP level of theory from the corresponding TS toward the product (Fig. S7). SCS-MP2 was chosen as the reference method based on literature confirming its accuracy in TS geometries and energies for DA reactions. [24]

All calculations were performed using the ORCA (version 4.2.1).[25] TS optimisation and single-point calculations were carried out with "Tight" convergence criteria. DFT functionals considered here included B2PLYP-D3BJ [26][19], M06-2X [27] (tested with and without D3BJ dispersion), ωB97M-D3BJ [28], ωB97X-D3 [29] and ωB97X-D3BJ. [30] All DFT calculations employed resolution-of-identity chain-of-spheres exchange (RIJCOSX) approximation.[31] The def2-TZVP [32] basis set was used in all calculations, and Coulomb integrals were approximated using the def2/J auxiliary basis set. [33] Among the tested DFT functionals, the ωB97M-D3BJ/def2-TZVP level of theory yielded the most accurate TS geometries (Table S5, RMSD =0.042 Å), as well as energy differences between TS and products(Fig. S7, RMSE = 0.95 kcal mol^{-1}). Therefore, this level of theory was selected as the ground-truth method for training the MLP.

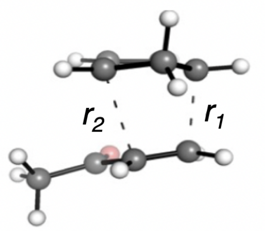

Table S5: Comparison of TS geometries between various DFT functionals and SCS-MP2. Δr is the length difference between the forming carbon bonds, r_1 and r_2. Root Mean Square Deviations (RMSD) between reference and DFT geometries for heavy atoms. All distances are in Å. The values in bold have the best agreement with the SCS-MP2.

	r_1	r_2	Δr /Å	$\Delta r/(r_1 + r_2)$	RMSD
SCS-MP2	2.099	2.438	0.339	0.075	—
B2PLYP D3BJ	2.033	2.509	0.476	0.105	**0.023**
M06-2X	2.001	**2.458**	0.457	0.102	0.071
M06-2X-D3BJ	1.998	**2.465**	0.467	0.105	0.061
ωB97M-D3BJ	**2.057**	2.360	**0.303**	**0.069**	**0.042**
ωB97X-D3	1.999	2.552	0.553	0.121	0.048
ωB97X-D3BJ	**2.068**	2.392	**0.324**	**0.073**	0.043

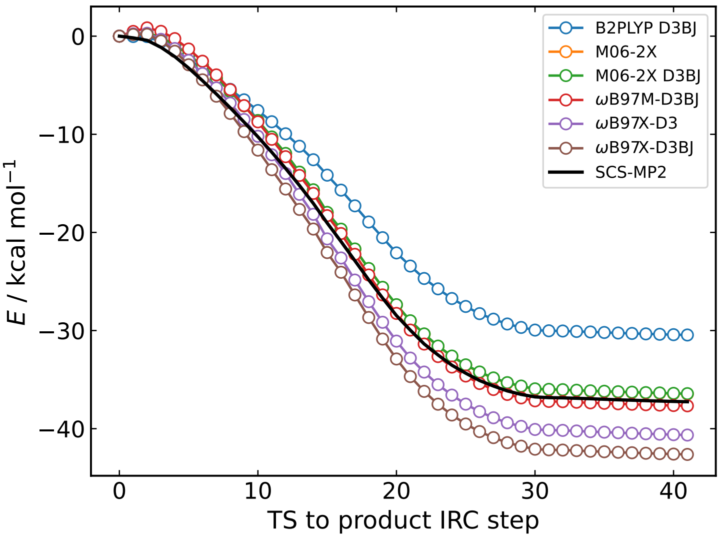

Figure S7: Energy comparisons for SCS-MP2 and selected DFT functionals. Single-point energies performed along the IRC generated by the PBE0-D3BJ/def2-SVP method from TS to the product of the reaction.

S5 The Accuracy of ACE MLPs in Implicit Solvents

ACE MLPs were trained by AL using the initial configurations of the gas-phase TS. The ground-truth DFT was the same as in gas phase computations, with the Conductor-like Polarizable Continuum Model (CPCM) [34] included to capture the solvation effects. To evaluate the ACE MLPs' accuracy, 200 fs ACE MLP-MD simulations were conducted, followed by a point-to-point comparison of energies and forces between the ACE MLPs and the ground-truth method for the structures obtained from the trajectories. The results are illustrated in figures S8, S9, S10 and S11. The simulation time was shorter than in explicit solvents (500 fs) as the reactions typically reached the reactant or product state within 200 fs of MD simulation.

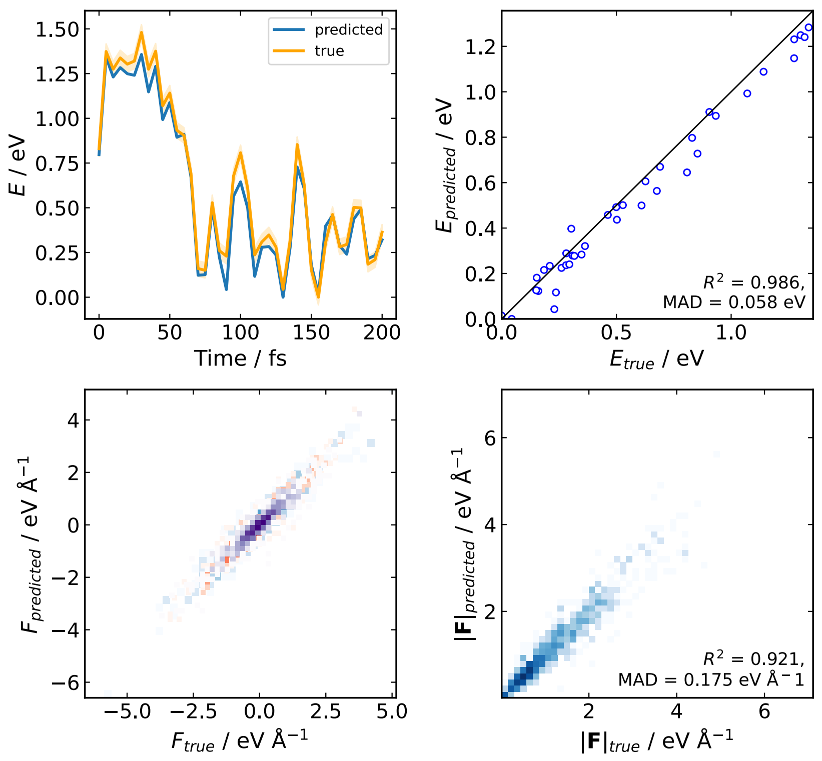

Figure S8: Performance of the ACE MLP for the reaction of CP + MVK in implicit water. Comparisons of ground-truth (CPCM(water)-ωB97M-D3BJ/def2-TZVP) and predicted (ACE MLP) energies and forces over 200-fs ACE MLP-MD trajectories from gas-phase TS (300 K, time step = 0.5 fs) for *endo* reaction.

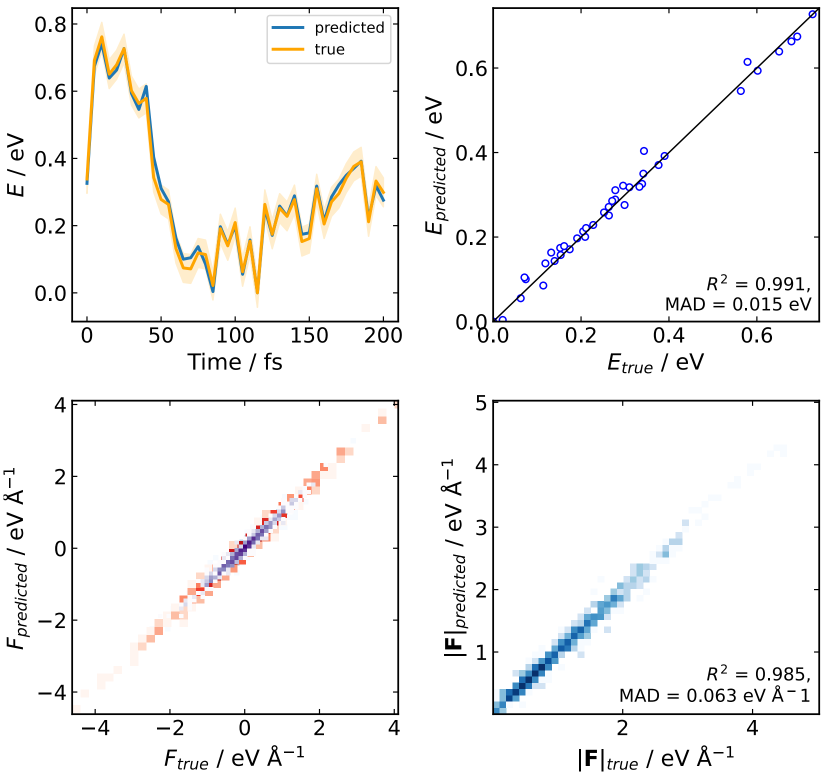

Figure S9: Performance of the ACE MLP for the reaction of CP + MVK in implicit water. Comparisons of ground-truth (CPCM(water)-ωB97M-D3BJ/def2-TZVP) and predicted (ACE MLP) energies and forces over 200-fs ACE MLP-MD trajectories from gas-phase TS (300 K, time step = 0.5 fs) for *exo* reaction.

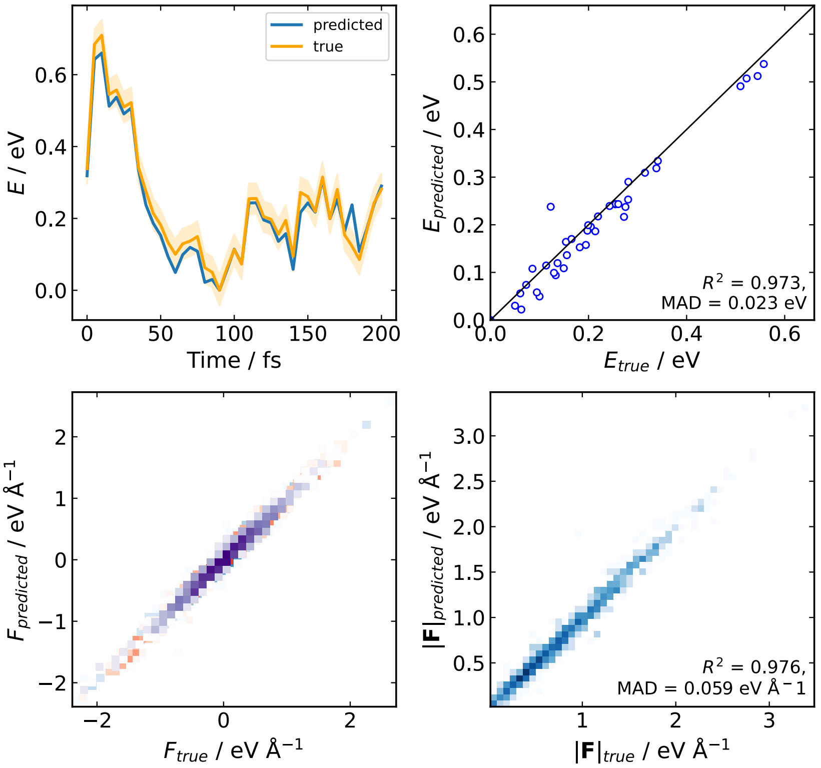

Figure S10: Performance of the ACE MLP for the reaction of CP + MVK in implicit methanol. Comparisons of ground-truth (CPCM(methanol)-ωB97M-D3BJ/def2-TZVP) and predicted (ACE MLP) energies and forces over 200-fs ACE MLP-MD trajectories from TS (300 K, time step = 0.5 fs) for (a) *endo* reaction.

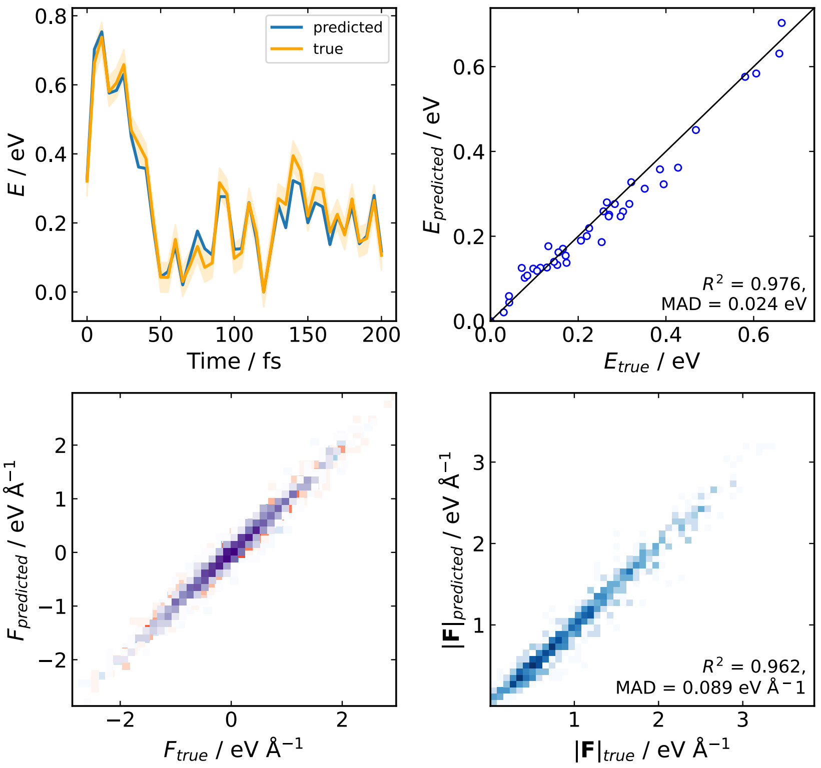

Figure S11: Performance of the ACE MLP for the reaction of CP + MVK in implicit methanol. Comparisons of ground-truth (CPCM(methanol)-ωB97M-D3BJ/def2-TZVP) and predicted (ACE MLP) energies and forces over 200-fs ACE MLP-MD trajectories from TS (300 K, time step = 0.5 fs) for *exo* reaction.

S6 Accuracy of ACE MLPs in Explicit Solvents

S6.1 Training Strategy for ACE MLPs

The overall training set consists of four subsets, each aimed at describing different interactions in the reactive system (Table S6). Subset 1 corresponds to the structures generated from the transition state (TS) of the system in vacuum (CP + MVK) and provides information about intramolecular interactions and intrinsic reactivity. Subset 2 consists of the structures generated from the same gas-phase TS, this time microsolvated with either two water molecules (as shown in Fig. 3(a) in the main text) or one methanol molecule, depending on the solvent used. The solvent molecules were randomly placed around the solute carbonyl group, constrained to form hydrogen bonds. Subset 3 corresponds to the gas-phase TS structure now fully solvated with 33 explicit water or methanol molecules. Subsets 2 and 3 aim to describe various solute-solvent interactions occurring during the reaction. Finally, subset 4 contains only solvent molecules (27 water or methanol molecules), providing information about solvent-solvent interactions in bulk solvent.

Table S6: Number of configurations obtained after AL and the final training set selected for different reactions

Subset	Initial structure for training	Reactions	# Configs.	
			After AL	Selected
1	gas-phase TS of CP+ MVK ($TS_{\mathrm{CP+MVK}}$)	*endo* in water	215	150 for each
		exo in water	229	
		endo in methanol	215	
		exo in methanol	229	
2	$TS_{\mathrm{CP+MVK}}$ + 2 water/1 methanol	*endo* in water	230	150 for each
		exo in water	258	
		endo in methanol	215	
		exo in methanol	235	
3	$TS_{\mathrm{CP+MVK}}$ + 33 solvent molecules	*endo* in water	260	250 for each
		exo in water	259	
		endo in methanol	252	
		exo in methanol	255	
4	pure solvent, either 27 water or methanol molecules	*endo* in water	52	50 for each
		exo in water	52	
		endo in methanol	81	
		exo in methanol	81	

All subsets were generated independently using the AL scheme (Fig. S12(a)) with the structure used to initiate the training listed in Table S6, as outlined in Fig. S12(b). Since one structure is

not enough to train the first version of the MLP, we generated 10 initial configurations as follows. For subsets 1 and 2, initial configurations were generated by adding the random displacement to atomic positions of the gas-phase TS structure. Since the ACE potential requires information on the box size for all structures in the training set, subsets 1 and 2 were assigned a cubic box of 100 Å. For subsets 3 and 4, the 10 initial configurations were generated by randomly placing the solvent molecules in a cubic box of 11 and 9.32 Å, respectively, to ensure the water density was reproduced. For methanol, the box was adjusted according to its density of 792 kg m^{-3} at room temperature. To train the ACE potential and propagate dynamics in the AL, the size of the boxes was then set to 100 Å without changing the positions of the molecules to create a solvated cluster in the gas phase.

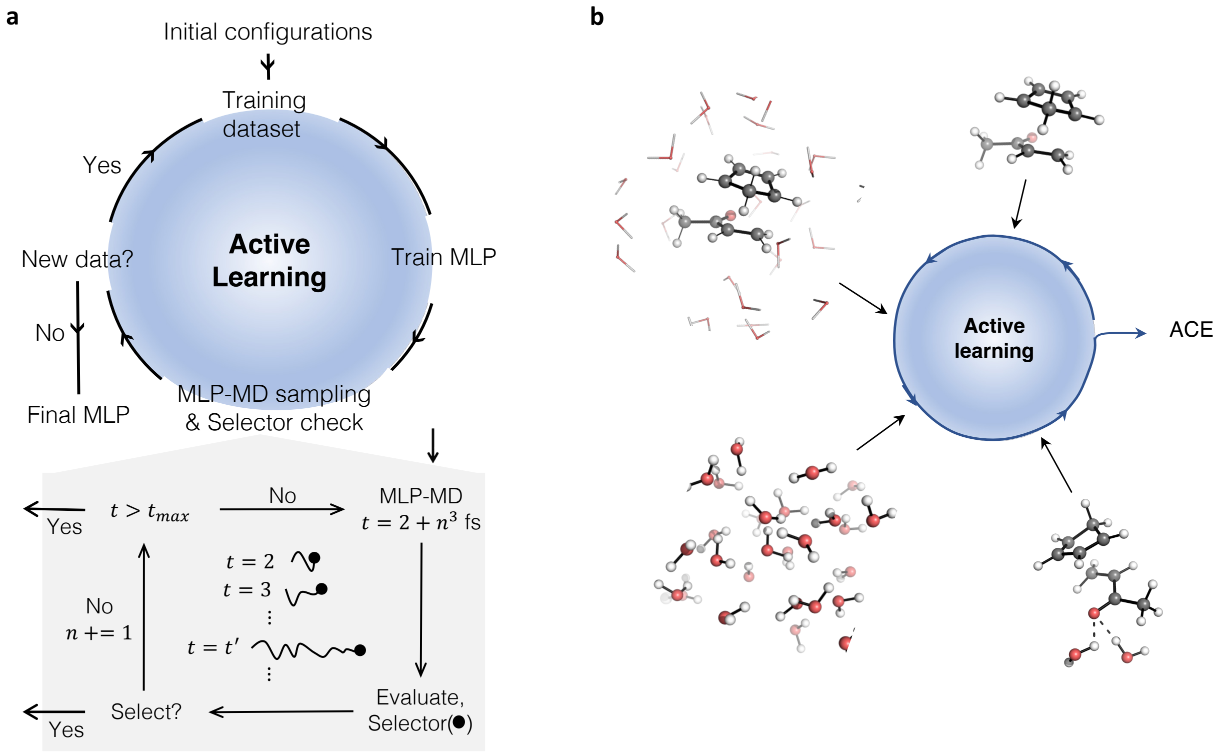

Figure S12: Training strategy implemented in this study. (a) Schematic representation of the active learning strategy. The grey box illustrates the detailed procedure of MLP-MD sampling and selector evaluation. (b) Generation of training data for the *endo* reaction in water, showcasing representative initial configurations for each subset.

For all subsets, the 10 initial configurations were used to train the first version of MLP. We trained one MLP for each of the subsets and used it to propagate short MLP-MD forward (product) or backward (reactants) from the TS and continue AL. The direction was assigned randomly based on the initial velocities from a Maxwell-Boltzmann distribution at 300 K. In all cases, MLP-MD was propagated for $(2 + n^3)$ fs, using a time step of 0.5 fs, with n (integer) initiated at 0. The chosen selector was then applied to evaluate the last frame of the trajectory to determine if the structures

would be added to the existing training set. If the selector did not find any new data, the value of n increased until it reached the maximum time of 5 ps. This approach ensures that the full reaction profile is sampled in the AL and that all relevant structures along the path are included in the data set. This simulation time was sufficient to sample the chemical and conformational space of the reaction. We acknowledge that for more complex systems, an adjustment in simulation time may be necessary, in particular for larger systems, where confirmation changes lead to several minima requiring longer times to reach the global minima in either direction.

After generating these four datasets by AL, they are combined by randomly selecting a specific number of configurations listed in Table S6 to train the final MLP. Each of the datasets obtained from the AL was reduced by 1 – 42 % to reduce the memory requirements for training the MLP on all data points, as ACE descriptor scales as S^{ν} (where S is the number of elements and ν represents the correlation number) for each data point. Data points from Subsets 1 and 2, corresponding to the reaction in the gas phase and microsolvated environment, were reduced by 30 – 35 % and 30 – 42 %, respectively. This decision is driven by the fact that the structural information provided by these subsets overlaps significantly. Data in subset 3 was reduced by only 1 – 4 % to ensure a proper description of solvent–substrate interactions. These reduced datasets were then combined, resulting in the final 600 configurations for training for each reaction. The combination of gas-phase and microsolvated data ensures that the training set contains information about the relevant states (RS, TS, PS) configurations along the reaction paths and relevant interactions for each of them. The use of cluster subsets in the training makes the process much more efficient compared to computationally more expensive periodic Density Functional Theory (DFT) calculations. Moreover, this approach enables the use of higher-level theory calculations, both in terms of method and basis sets. This is a limitation in periodic ab initio MD (AIMD) calculations, which are typically restricted to GGA or, at most, hybrid GGA functionals with specialized plane-wave basis sets.

S6.2 Reaction in Water

By utilising the AL training strategy described in SI §S6.1, we obtained MLPs to simulate the reaction in the explicit solvent. Before assessing the accuracy of the MLP, we analysed to which extent the training data covered the RS, TS, PS, and the pathways connecting them for the *endo* reaction in water. Fig. S13(a) illustrates the "closeness" of the training data to the 2D PES. The darker regions indicate the areas most similar to the data in the training set, while the pink regions indicate less covered areas. The white dashed line represents the reaction pathway obtained by a partial nudged elastic band (NEB). This path crosses a well-covered region of the PES, even though it is relatively far from the initial training point (highlighted by the white circle). Fig. S13(b) shows the exact positions of the training data points. The training data include RS configurations with a larger distance between the reactant than at the PES discussed below, confirming further that the AL method adequately sampled the reaction profile.

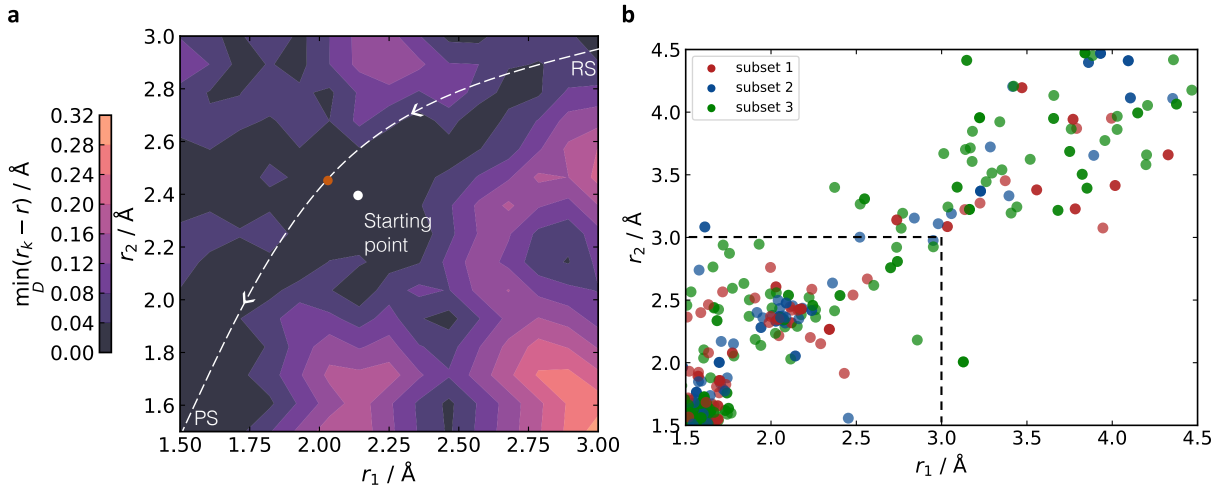

Figure S13: Closeness and locations of training data for the *endo* reaction in water. (a) The relative distance between each point in the training data and the reaction PES generated by the 2-D scan, measured by $\min(r_k - r)$, which represents the minimum distance, averaged between r_1 and r_2, between the point on the surface and all points in the training dataset. The darkest regions ($\min(r_k - r) = 0$) correspond to data in the training set, while pink/orange regions indicate areas less well-represented. The white circle corresponds to the gas phase TS, used as a starting point for AL. The white dashed line depicts the reaction path in explicit 200 water molecules, with the highest energy highlighted in orange. (b) The location of training data is colour-coded based on the origin of the data point. The region corresponding to the PES shown in (a) is enclosed by dashed lines.

The performance of the trained ACE MLP for the CP + MVK reaction in explicit water was evaluated in two different systems. The first one corresponds to the gas phase TS to which we added three water molecules that form hydrogen bonds (HBs) with the carbonyl group in MVK (illustrated on the left-hand side of Fig. S14 for *endo* reaction). This system was used to assess the accuracy of the MLP in describing hydrogen bonding interactions. The second system corresponds

to the gas phase TS immersed in a water box containing 55 water molecules (box size = 12.42 Å), as depicted on the left-hand side of Fig.4(b) in the Main text for *endo* reaction. This system was used to evaluate the ability of the ACE MLPs to model large systems. To assess the accuracy of ACE MLPs for DA reactions, 500 fs ACE MLP-MD simulations with a timestep of 0.5 fs were conducted in an NVT ensemble at 300 K using a Langevin thermostat. To validate whether the ACE MLP enables capturing the solvent arrangements, frames from a 3-ps *endo* ACE MLP-MD trajectory of the second system were collected and compared the accuracy of MLP and ground-truth method (Fig. S17).

In each case, the accuracy of ACE MLPs was demonstrated by comparing the ground truth and predicted energies and forces. The energy errors for both *endo* (Fig. S14, Fig. 3(b) and Fig. S17) and *exo* (Fig. S15 and S16) reactions in water were lower than 1 kcal mol^{-1}per molecule, while the force errors were around 2 kcal mol^{-1}Å^{-1}. The stability and transferability to PBC of the MLP were assessed by 50 ps NVE simulation (shown in Fig. S18).

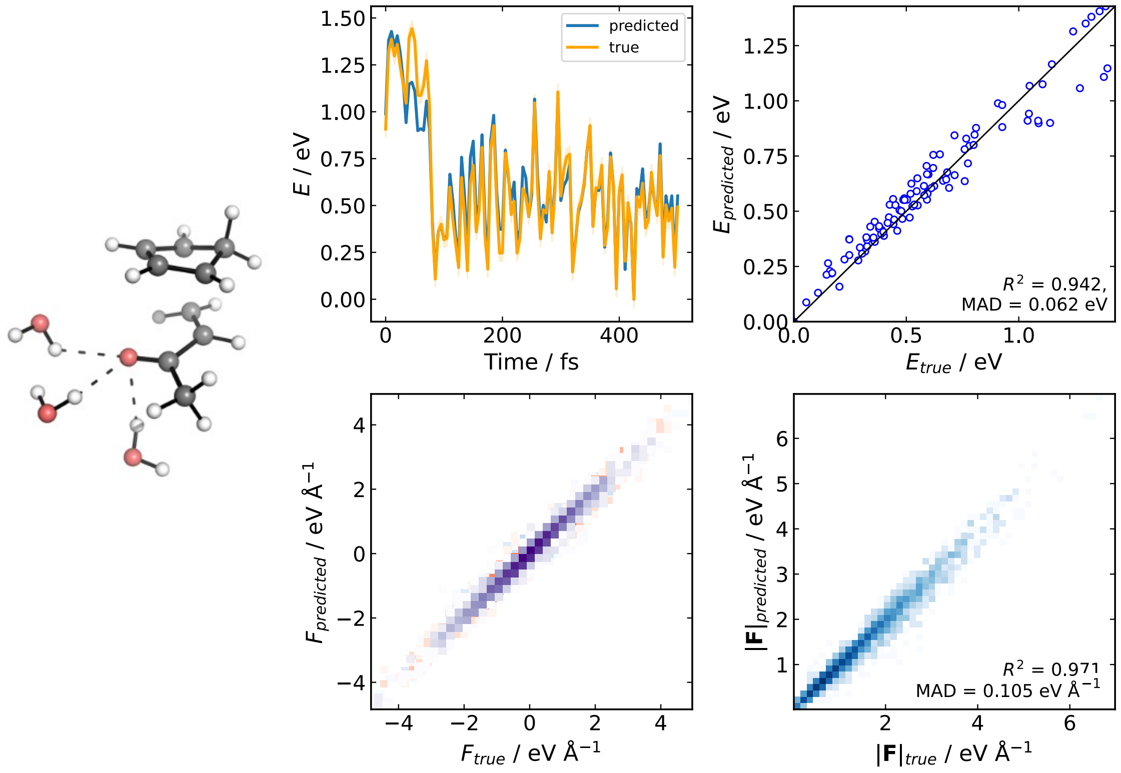

Figure S14: Comparisons of ground-truth (ωB97M-D3BJ/def2-TZVP) and predicted (ACE MLP) energies and forces for the *endo* reaction of CP + MVK in water. Values obtained for frames in a 500-fs ACE MLP-MD trajectory of gas-phase TS bounded to three water molecules (300 K, time step = 0.5 fs).

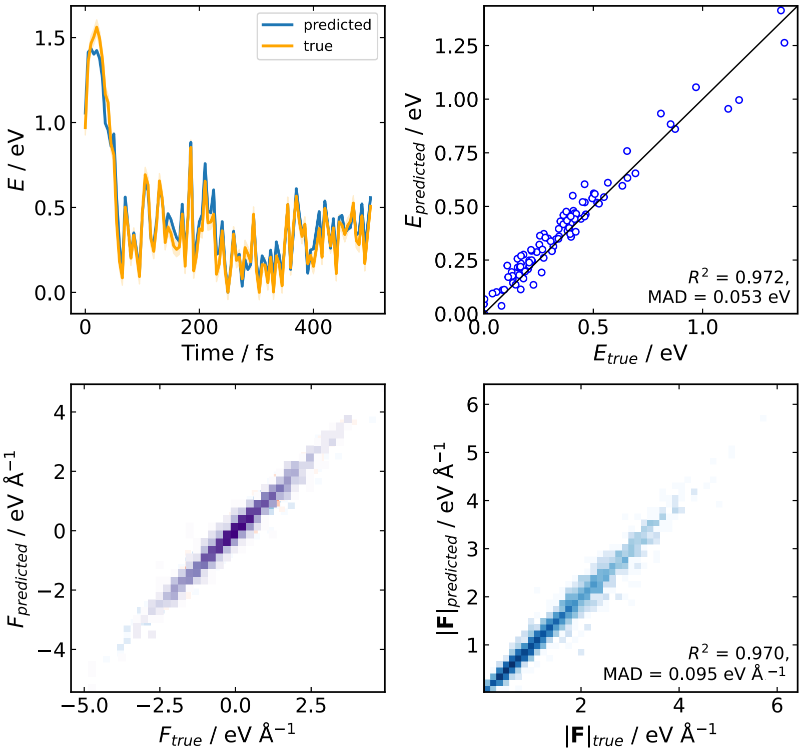

Figure S15: Performance of the ACE MLP for the *exo* reaction of CP + MVK in water. Comparisons of ground-truth (ωB97M-D3BJ/def2-TZVP) and predicted (ACE MLP) energies and forces during 500-fs ACE MLP-MD trajectories from gas-phase TS bounded to three water molecules (300 K, time step = 0.5 fs).

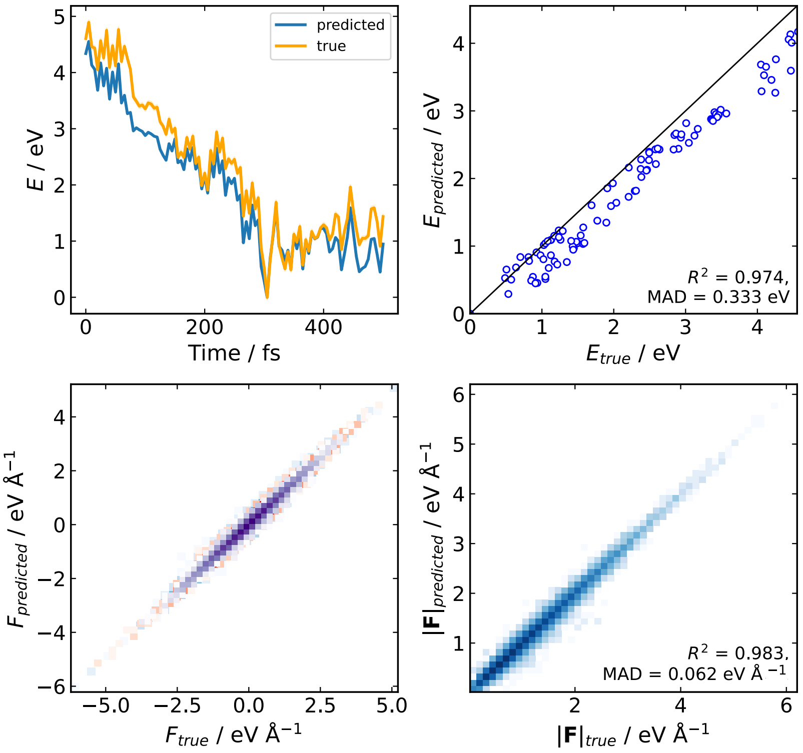

Figure S16: Performance of the ACE MLP for the *exo* reaction of CP + MVK in water. Comparisons of ground-truth (ωB97M-D3BJ/def2-TZVP) and predicted (ACE MLP) energies and forces during 500-fs ACE MLP-MD trajectories from gas-phase TS geometry immersed in 55 water molecules, which was equilibrated by 1 ps MD simulation with fixed TS before propagating (300 K, time step = 0.5 fs).

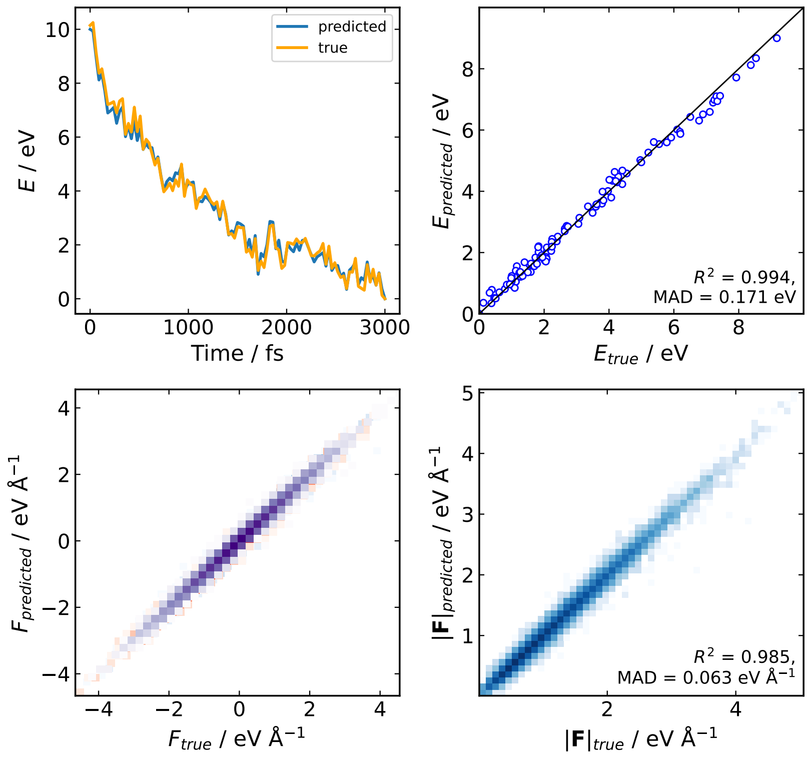

Figure S17: Performance of the ACE MLP for the *endo* reaction of CP + MVK in water for a 3-ps trajectory. Comparisons of ground-truth (ωB97M-D3BJ/def2-TZVP) and predicted (ACE MLP) energies and forces over an ACE MLP-MD trajectory started from TS (obtained in the gas phase) solvated in 55 water, which was equilibrated by 1 ps MD simulation with fixed TS before propagating (300 K, time step = 0.5 fs).

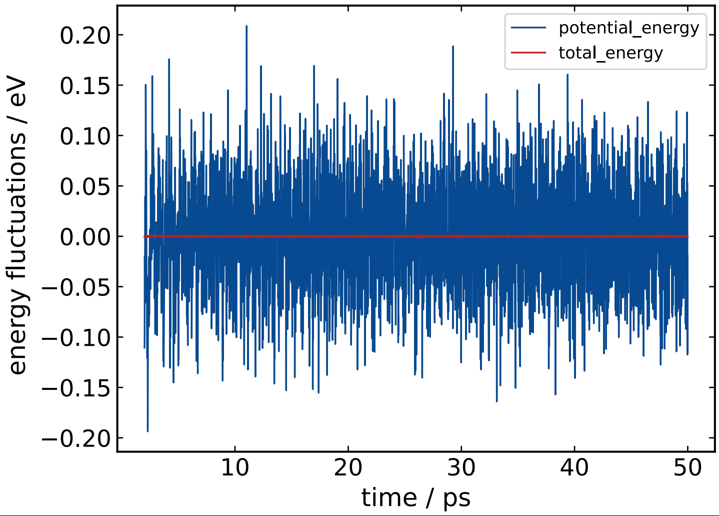

Figure S18: Energy fluctuations of the ACE MLP for the *endo* reaction of CP + MVK (reactant state) in 320 water during 50 ps NVE simulation under PBC

S6.3 Reaction in Methanol

The ACE MLP validation for the reaction in explicit methanol was performed for two different systems, similar to the ones for explicit water. The first system consisted of the gas-phase TS bounded to two methanol molecules (Fig. S19(a)). The number of solvent molecules was decreased due to the larger size of methanol molecules compared to water. The bulk methanol system consisted of the gas phase TS solvated in 40 methanol molecules (box size of 14.5 Å), illustrated in Fig. S19(b). To evaluate the ACE MLPs' accuracy for DA reactions, 500 fs MLP MD simulations with a timestep of 0.5 fs were conducted in an NVT ensemble at 300 K using a Langevin thermostat, followed by a point-to-point comparison of energies and forces between the ACE MLPs and the ground truth method for the structures obtained from the trajectories. Similar to the reaction in water, solvent arrangement was validated by a 3-ps *endo* MLP MD trajectory (Fig. S24).

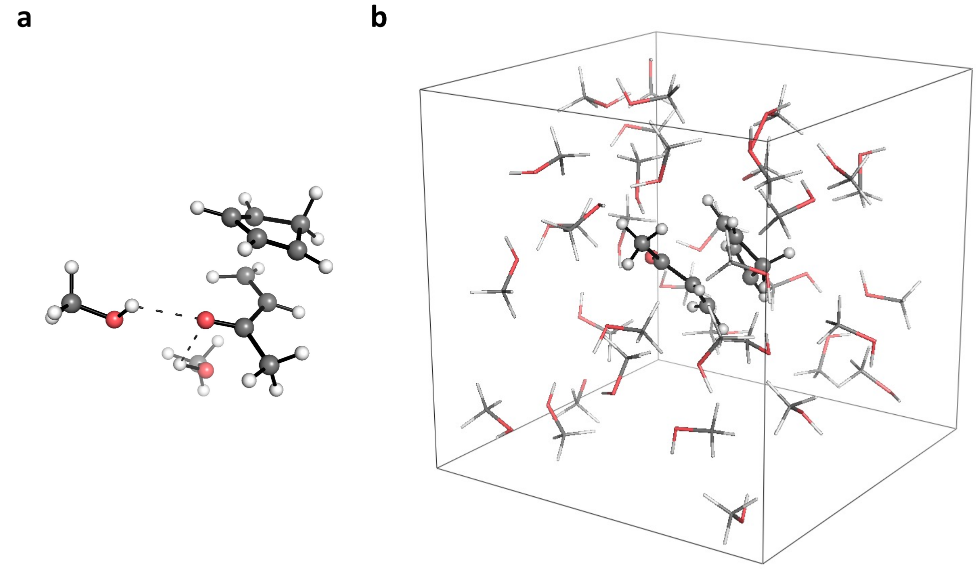

Figure S19: Systems tested to validate the trained ACE MLP for the reaction of CP + MVK in methanol. (a) gas-phase TS bounded to two methanol molecules. (b) Gas-phase TS immersed in a box with 40 methanol molecules, with a size of 14.5 Å.

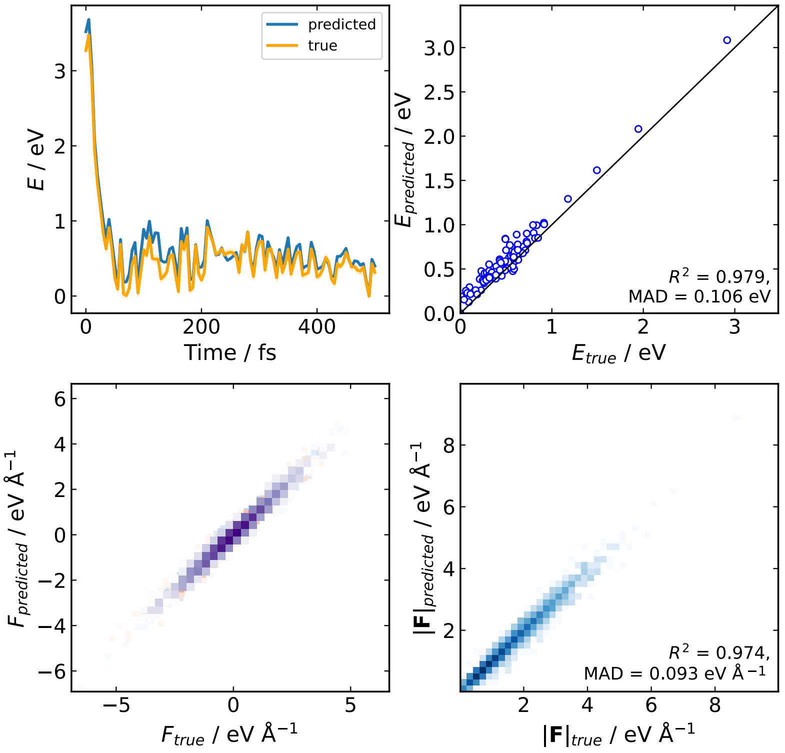

Figure S20: Performance of the ACE MLP for the *endo* reaction of CP + MVK in methanol. Comparisons of ground-truth (ωB97M-D3BJ/def2-TZVP) and predicted (ACE MLP) energies and forces over 500-fs ACE MLP-MD trajectories for gas-phase TS bounded to two methanol molecules(300 K, time step = 0.5 fs).

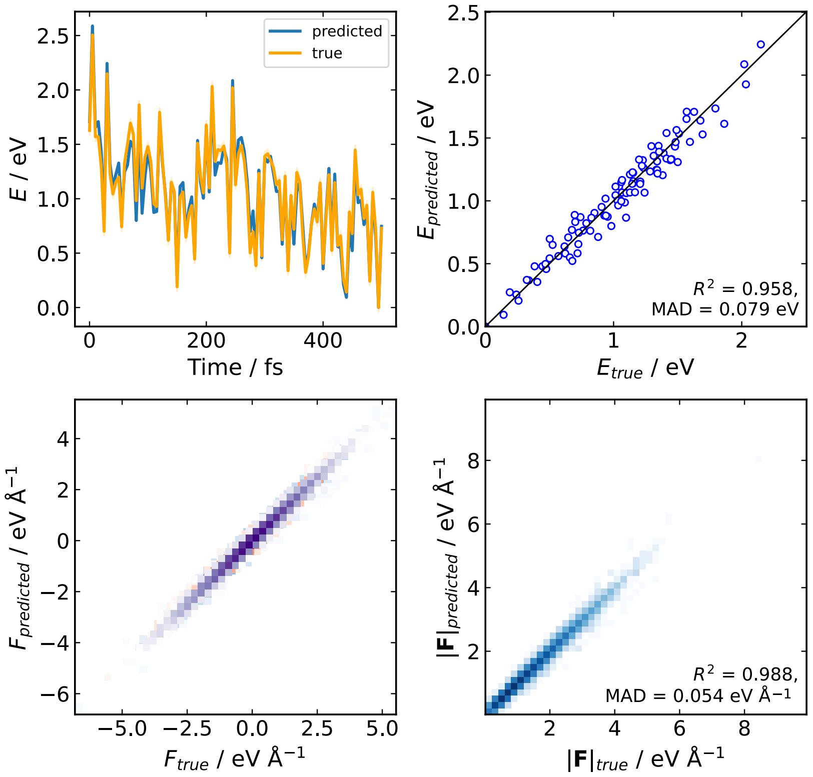

Figure S21: Performance of the ACE MLP for the *endo* reaction of CP + MVK in methanol. Comparisons of ground-truth (ωB97M-D3BJ/def2-TZVP) and predicted (ACE MLP) energies and forces over 500-fs ACE MLP-MD trajectories for TS (obtained in the gas phase) solvated in 40 methanol molecules, which was equilibrated by one ps MD simulation with fixed TS before propagating (300 K, time step = 0.5 fs).

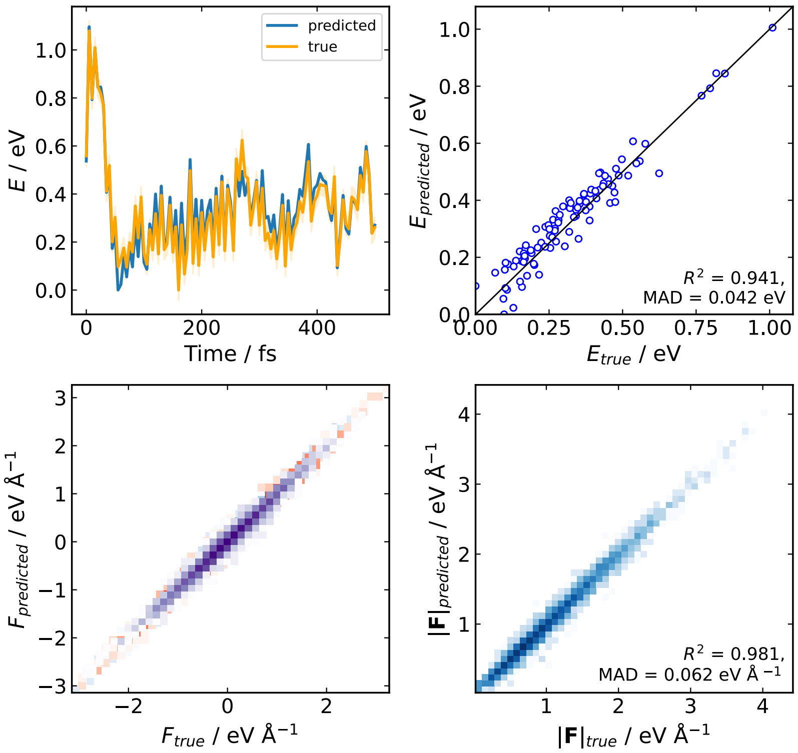

Figure S22: Performance of the ACE MLP for the *exo* reaction of CP + MVK in methanol. Comparisons of ground-truth (ωB97M-D3BJ/def2-TZVP) and predicted (ACE MLP) energies and forces over 500-fs ACE MLP-MD trajectories for gas-phase TS bounded to two methanol molecules (300 K, time step = 0.5 fs).

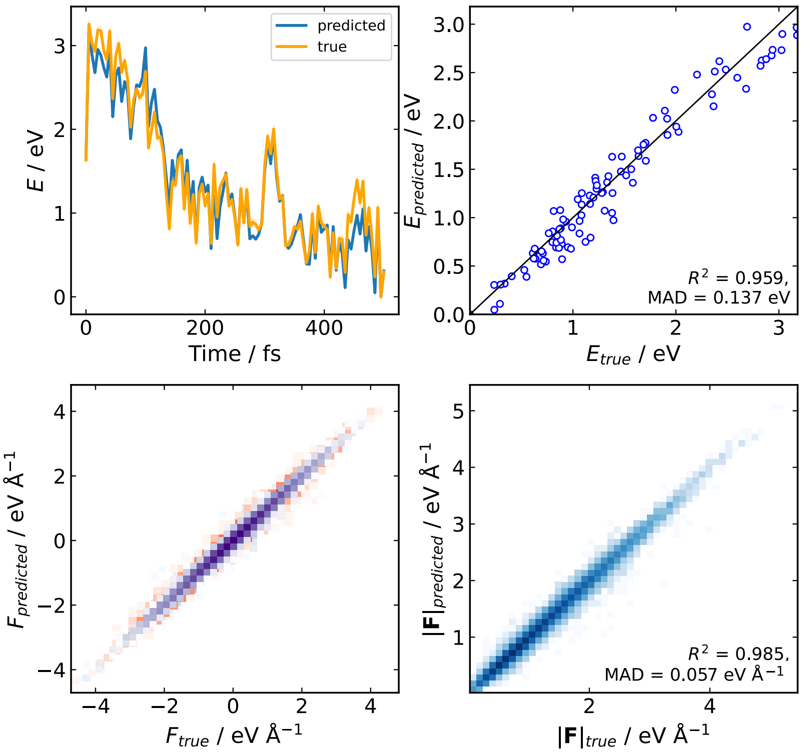

Figure S23: Performance of the ACE MLP for the *exo* reaction of CP + MVK in methanol. Comparisons of ground-truth (ωB97M-D3BJ/def2-TZVP) and predicted (ACE MLP) energies and forces over 500-fs ACE MLP-MD trajectories for gas-phase TS geometry solvated in 40 methanol molecules, which was equilibrated by one ps MD simulation with fixed TS before propagating(300 K, time step = 0.5 fs).

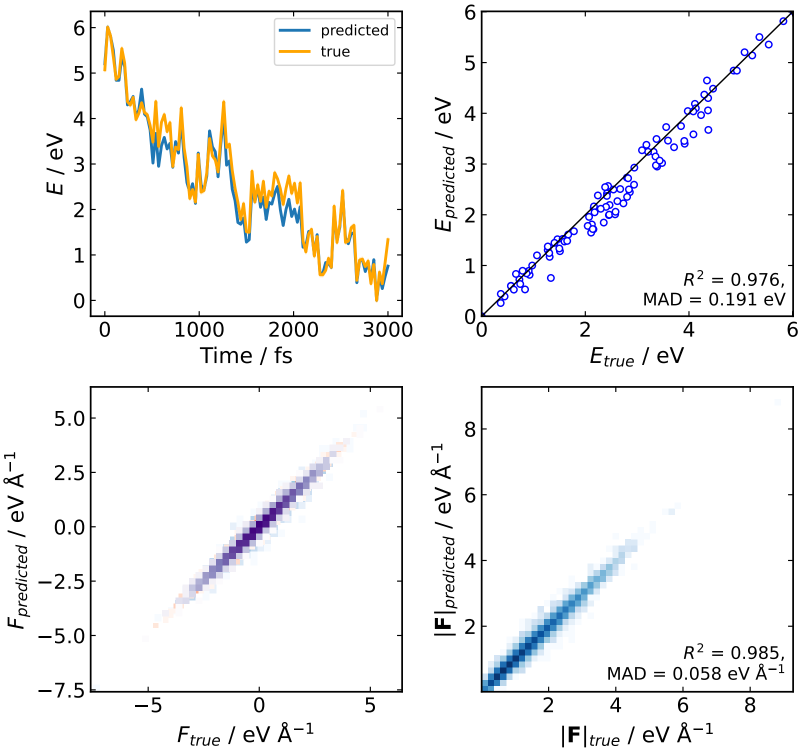

Figure S24: Performance of the ACE MLP for the *endo* reaction of CP + MVK in methanol for a 3-ps trajectory. Comparisons of ground-truth (ωB97M-D3BJ/def2-TZVP) and predicted (ACE MLP) energies and forces over an ACE MLP-MD trajectory started from TS (obtained in the gas phase) solvated in 44 methanol, which was equilibrated by 1 ps MD simulation with fixed TS before propagating (300 K, time step = 0.5 fs).

S6.4 Summary of the explicit solvent MLPs accuracy

In SI §S6.2-6.3, we compared MAD for two systems with different compositions than the training data. Here, we extend the validation to systems with the same composition as the training data, referred to as training-like systems. All data used for testing were generated separately from the training to prevent data leakage and unforeseen extrapolation errors. Each of the four final MLPs (i.e., one for endo and exo reaction in water and methanol solvents) was evaluated over 4 sets of structures containing 101 structures each, generated by independent 500-fs ACE MLP-MD trajectories

In the table below, we summarise MAD for energy and forces across all the tested systems, i.e., the previously reported testing system and newly generated training-like systems. For sets 1 and 2 in Table S7 and S8, the simulations were initiated from structures with randomly placed solvent molecules. The resulting dynamics thus included the equilibration of solvent molecules, as evident from the rapid decrease in energy shown in Figs. S14, S15, S20 and S22. The energy and force errors in these systems range from 1.27 meV atom^{-1} to 3.56 meV atom^{-1} and 62 meV Å^{-1} to 116 meV Å^{-1}, respectively. In the second case, i.e., sets 3 and 4 in Table S7 and S8, solvent molecules were equilibrated using MLP-MD before running the validation MD. The energy and force errors for water and methanol for solvated systems vary from 0.3 meV atom^{-1} to 1.8 meV atom^{-1} and 52 meV Å^{-1} to 62 meV Å^{-1}. These validations indicate that adding more solvent molecules does not deteriorate the accuracy and the potentials have sufficient transferability across systems with different sizes. The large errors for testing in entries 1 and 2 in Table S7 and S8 are likely due to distorted structures in the validation trajectory, as these MDs were initiated from randomly placed solvent molecules. While testing MLPs on trajectories starting from randomly placed molecules may seem counterintuitive, this approach challenges the performance of MLPs in modelling distorted structures and out-of-equilibrium geometries.

Table S7: Errors of ACE MLPs for Diels-Alder reaction in water and methanol with testing system larger than training systems. Each set contains 101 structures generated by independent 500-fs ACE MLP-MD trajectories.

	Testing System	Water		Methanol	
		Energy MAD (meV atom^{-1})	Force MAD (meV Å^{-1})	Energy MAD (meV atom^{-1})	Force MAD (meV Å^{-1})
1	TS_{CP+MKV} + 3 H_2O / 2 CH_3OH, *endo*	2.07	105	3.21	93
2	TS_{CP+MKV} + 3 H_2O / 2 CH_3OH, *exo*	1.77	95	1.27	62
3	TS_{CP+MKV} + 55 H_2O / 40 CH_3OH, *endo*	0.37	59	0.30	54
4	TS_{CP+MKV} + 55 H_2O / 40 CH_3OH, *exo*	1.79	62	0.52	57

Table S8: Errors of ACE MLPs for the Diels-Alder reaction in water and methanol for different training-like systems. Subset 2 consists of TS CP + MVK + 2 water /1 methanol molecule(s), while Subset 3 consists of TS CP + MVK + 33 solvent molecules. The configurations used to test the MLPs were generated independently by ACE MLP-MD and were not included in the training data.

	Training-like System	Water		Methanol	
		Energy MAD (meV atom^{-1})	Force MAD (meV Å^{-1})	Energy MAD (meV atom^{-1})	Force MAD (meV Å^{-1})
1	Subset 2, *endo*	2.50	88	1.61	116
2	Subset 2, *exo*	1.75	77	3.56	106
3	Subset 3, *endo*	0.48	58	0.68	52
4	Subset 3, *exo*	0.97	58	0.86	56

S7 Reaction Coordinate

The reaction coordinate chosen in this study corresponds to the average bond length of the two C-C bonds formed during the reaction, denoted as $\bar{r} = (r_1 + r_2)/2$ (Figure S25). The impact of the selected reaction coordinates on the 1D free energy surfaces for DA reactions has been previously discussed by Jorgensen *et al.* [35] It has been noted that if the reaction coordinate is not chosen appropriately, the TS obtained from the free energy surface may deviate significantly from the actual TS. This emphasizes the importance of carefully selecting the reaction coordinates to ensure accurate determination of the TS.

To validate the reaction coordinate, ACE MLP-MD/US simulations were performed for the *endo* reaction in the gas phase. From the resulting 1D free energy profile, we extracted the 1206 configurations with the highest energy and analyzed their C-C bond distance distribution (r_1, r_2). Representative configurations (243) from the highest probability bond lengths are considered to represent well the TS geometry. These configurations were compared with the TS obtained from the ground truth DFT functional and found to show good agreement. Specifically, the bond lengths obtained from the ACE MLP-MD/US simulations were 2.38 ± 0.010 Å and 2.10 ± 0.011 Å, while the ground truth DFT bond lengths were 2.36 Å and 2.06 Å, respectively. This agreement between the two sets of bond lengths provides evidence for the accuracy and suitability of the selected reaction coordinate for our study.

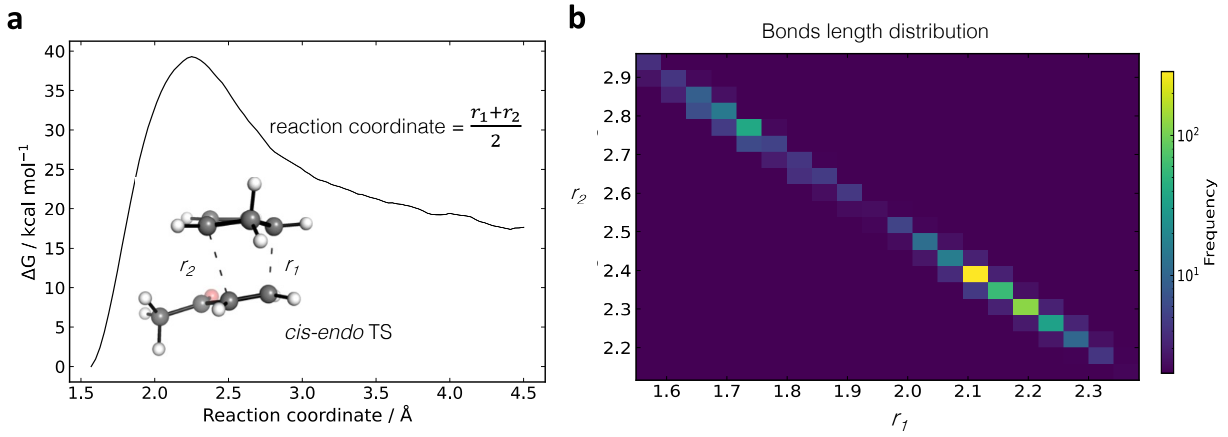

Figure S25: Reaction coordinate (a) 2D Free energy surface for the *endo* reaction in the gas phase calculated by ACE MLP-MD/US at 300 K with the reaction coordinate $\bar{r} = (r_1 + r_2)/2$. (b) Bond length distribution for configurations corresponding to the highest free energy.

S8 2D PES

Relaxed 2D potential energy surfaces (PESs) were generated using the trained ACE MLPs in implicit and explicit solvents along the r_1 and r_2 coordinates. As shown in Fig. 4 of the Main text, the 2D scan exhibits a broader, flatter area after the TS in explicit solvent compared to the implicit model. The edges of this area correspond to structures with one fully formed C-C bond with a bond length smaller than 1.6 Å and another not yet within a range of 2.5 to 3.0 Å, indicating the possible formation of zwitterionic or diradical species. To characterise the electronic structure of the species, we calculated the total spin expectation value $\langle S^2 \rangle$ of structures in this region with UωB97M-D3BJ/def2-TZVP, with multiplicity set to 1. The value of $\langle S^2 \rangle$ converged to zero, demonstrating the absence of diradical character and confirming the formation of zwitterionic species (ZS). Furthermore, we did not observe any spin contamination which would indicate a complicated electronic structure. As the ACE MLP were trained solely on energy and forces, they cannot provide direct information on the electronic structure. Instead, the species formed in the MLP dynamics reflect the stability at the DFT level.

In this study, we observed a formation of ZS intermediates during the reaction in explicit water. Similar effects as to the transient formation of the zwitterion were also reported for double proton transfer in formic acid in solution.[36] More detailed discussions on these findings can be found in Sections S9 and S10.1. These intermediates are denoted by a cross (triangle) for the *endo* (*exo*) intermediate in the following PES scans. To compare the stabilisation of the ZSs in different solvent environments, we computed the energies of these two structures with respect to the reactant state (RS).

S8.1 Implicit solvation

Comparison of the PES for the *endo* reaction in implicit water and methanol (Table S9) reveals that the ZS marked by the cross symbol in Fig. S26 is 6.7 kcal mol^{-1} more stable in water than in methanol, likely due to the higher polarity in water which better stabilises the ZS species.[37] Furthermore, the *exo* intermediates in both environments (marked by the triangle symbol in Fig. S26) exhibit significantly higher energies compared to the *endo* intermediates (the cross symbol).

Table S9: Energies (in kcal mol^{-1}) of the cross labelled (representing the intermediate for the *endo* reaction in explicit water) and the triangle labelled (representing the intermediate for the *exo* reaction in explicit water) zwitterionic species relative to the RS in different PESs. The PESs include *endo* with implicit water and methanol, as well as *exo* with implicit water and methanol.

Reaction/Environment	Water		Methanol	
	endo	*exo*	*endo*	*exo*
cross labelled species	8.9	19.1	15.6	19.4
triangle labelled species	34.0	35.3	34.3	35.7

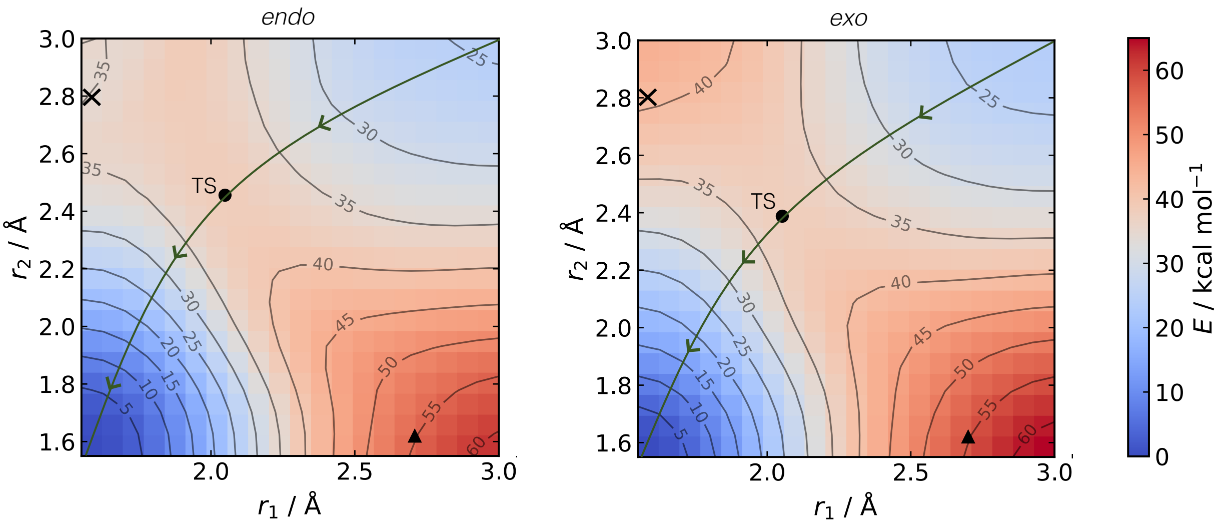

Figure S26: Relaxed 2D PES for *endo* and *exo* reaction of CP + MVK in implicit water along the r_1 and r_2 coordinates generated by the ACE MLPs. The solid lines represent the reaction pathway obtained from the ACE MLP static scan. The cross symbol indicates the intermediate for the *endo* reaction in explicit water, while the triangle symbol represents the intermediate for the *exo* reaction in explicit water.

S8.2 Explicit solvation

For *exo* reaction in explicit water, the position of ZS intermediate observed in PES is far away from that of *endo* reaction. Before delving into further details of the *exo* reaction in explicit water, it is necessary to further validate our ACE MLP for this reaction. In addition to the comparison of point-to-point energies and forces described in Section S6, we evaluated its dependence on the training data using the closeness metric,[16] which indicates how closely our training data aligns with the sampled configurations in the PES. Our results show that the intermediate region is well-sampled, indicating that the presence of this state is reliable and not an artefact of our method.

The existing reaction intermediates (represented by the cross and triangle labelled species for *endo* and *exo* in explicit water, respectively) show an increase in energies relative to the RS when considering explicit methanol as the solvent. This destabilisation of the intermediates in explicit methanol helps to explain the mechanistic differences observed for the reactions in water.

A comparison of the energies of labelled ZSs between implicit and explicit solvents (Table S9 and Table S10, respectively) reveals that explicit solvent provides greater stabilization for the ZSs. With explicit solvent, the *endo* ZSs (cross) experience an energy reduction of approximately 2.2-8.4 kcal mol^{-1} compared to the implicit solvent. Moreover, the ZSs for *exo* reaction (triangle) are stabilized by more than 10 kcal mol^{-1} when compared to the implicit model. This enhanced stabilization is likely attributed to non-covalent interactions, including electrostatic interactions and hydrogen bonding between the substrate and solvent molecules, which the implicit solvent model does not adequately capture.

Table S10: Energies (in kcal mol^{-1}) of the cross labelled (representing the intermediate for the *endo* reaction in explicit water) and the triangle labelled (representing the intermediate for the *exo* reaction in explicit water) zwitterionic species relative to the RS in different PESs. The PESs include *endo* with explicit water and methanol, as well as *exo* with explicit water and methanol.

Reaction/Environment	Water		Methanol	
	endo	*exo*	*endo*	*exo*
cross labelled species	6.7	10.7	12.1	12.9
triangle labelled species	18.1	9.2	17.7	15.5

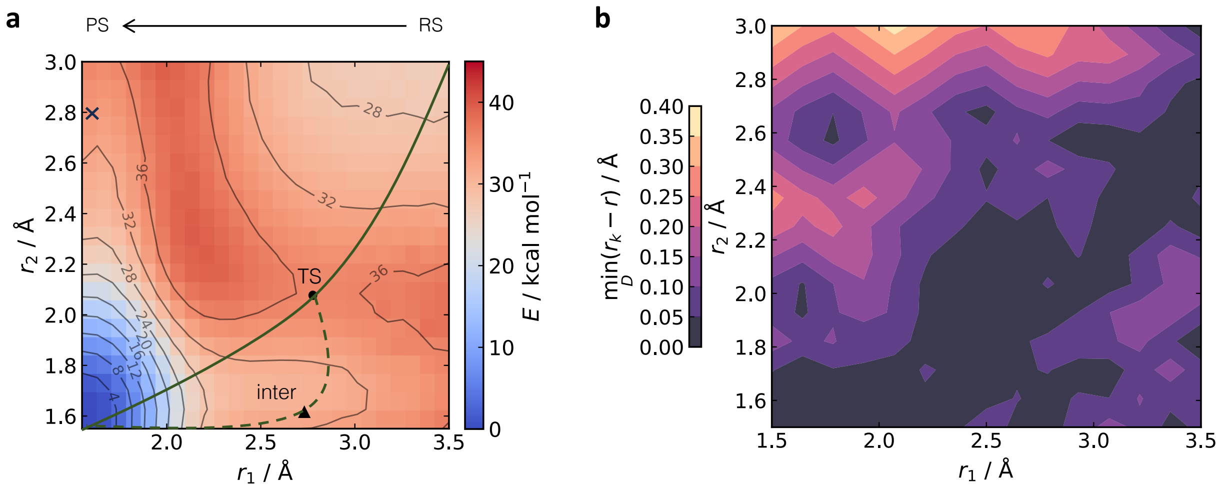

Figure S27: 2D PES and training data sampling. (a) Relaxed 2D PES scan along the r_1 and r_2 coordinates generated by the ACE MLP for the *exo* reaction of CP + MVK in explicit water (box size: 18.5 Å). The solid and dashed lines represent the reaction pathway obtained from the ACE MLP static scan and ACE MLP-MD, respectively. The cross symbol indicates the intermediate for the *endo* reaction in explicit water, while the triangle symbol represents the intermediate for the *exo* reaction in explicit water. (b) Visualisation of the "closeness" of the training data used to train the ACE MLP for the *exo* reaction of CP + MVK in explicit water.

S9 Free energy profile

The free energy calculations for *endo*/*exo* reactions were performed in explicit water and methanol using ACE MLP-MD/US simulations. The substrates CP and MVK were immersed in a box with a box size of 18.5 Å containing either 201 water or 90 methanol. The free energy profiles were computed in three repetitions to ensure convergence of the energy (Table S11). Errors were obtained from the standard deviation of the three repeats.

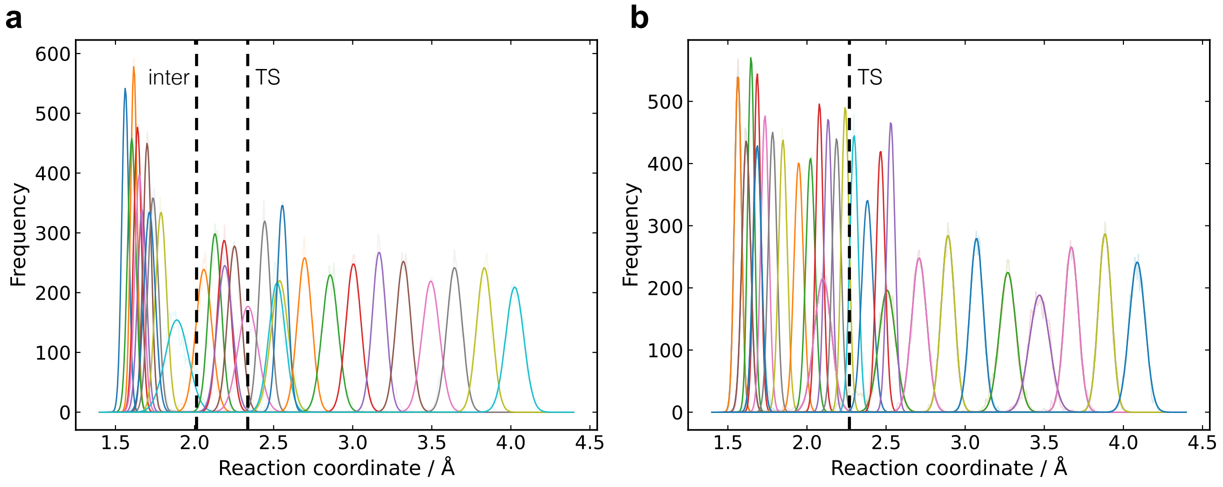

Figure S28: Histograms of the reaction coordinate for the *endo* reaction in (a) explicit and (b) implicit water. The positions of TS and intermediate (if existed) in the reaction coordinate are highlighted by dashed black lines.

Table S11: Activation free energy (kcal mol^{-1}) from ACE MLP-MD/US in different solvents. Experimental data (if available) is quoted in parentheses.

Reaction/Environment	Water		Methanol	
	Implicit	Explicit	Implicit	Explicit
endo	21.2±0.2 (19.2)	18.8±0.6 (19.2)	24.9±0.2(21.6)	20.5±0.8(21.6)
exo	23.6±0.4 (21.1)	20.3±0.8 (21.1)	24.7±0.1	21.5±0.7

From the TS we obtained from the free energy, we calculated the synchronicity of *endo* and *exo* reactions in different environments as listed below:

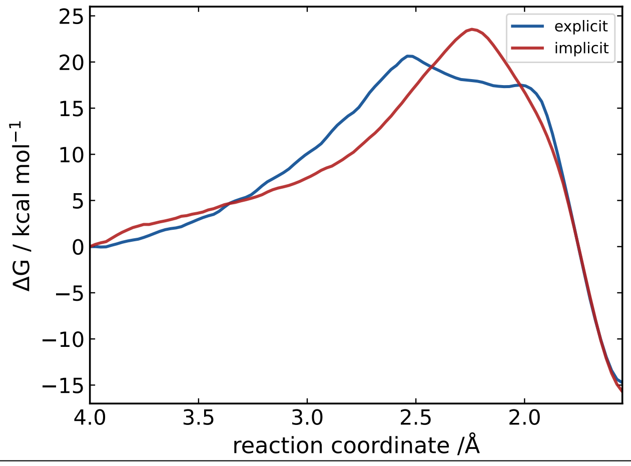

Figure S29: Free energy profiles obtained from ACE-MD/US for the *exo* reaction in implicit and explicit water.

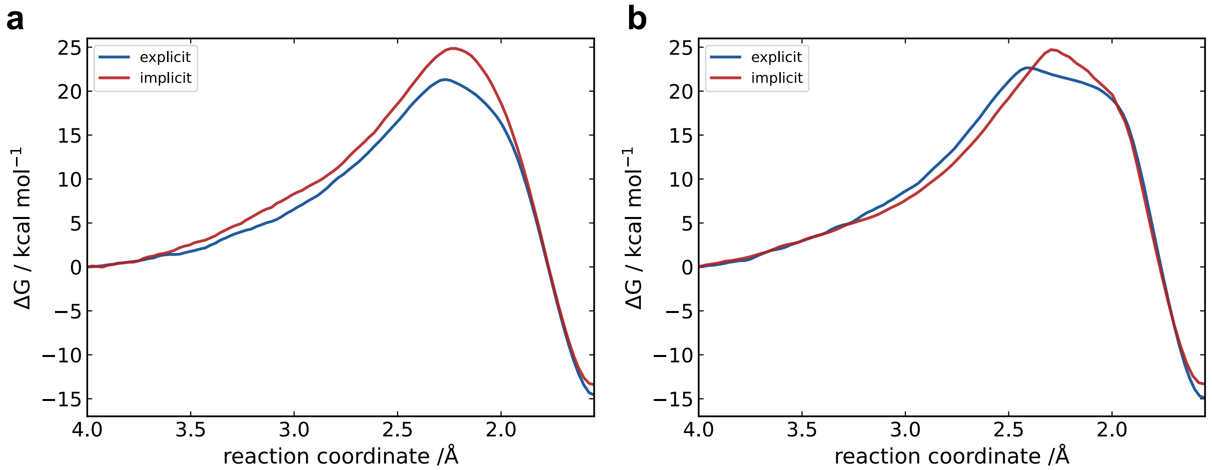

Figure S30: Free energy profiles obtained from ACE-MD/US of the (a) *endo* and (b) *exo* reaction in implicit and explicit methanol.

Table S12: synchronicity ($\Delta r = |r_2 - r_1|$, pm) for the *endo* and *exo* reactions in different solvents

Reaction/Environment	Gas	Water		Methanol	
		Implicit	Explicit	Implicit	Explicit
endo	30	37	46	40	7
exo	24	28	49	32	13

S10 Dynamics Studies

S10.1 Downhill Dynamics

The downhill dynamics simulations were initiated from the TS obtained from ACE-MD/US free energy calculation in explicit solvent and propagated without any constraints forward to product states (PS) and backward to RS. A trajectory was considered to react to the PS if both r_1 and r_2 were below 1.6 Å, whereas the RS was reached when both r_1 and r_2 exceeded 3.0 Å. We performed 500 downhill trajectories in total.

Table S13: Summary of 500 downhill trajectories initiated from the TS structures in explicit water with PBC and NVT ensemble (300 K, time step = 0.5 fs)

	# reactant	# product	Average time gap	# product with time gap < 60 fs
endo	228 (46%)	269 (54%)	84.3 fs	143 (53% of # product)
exo	212 (41%)	282 (56%)	130.7 fs	76 (27% of # product)

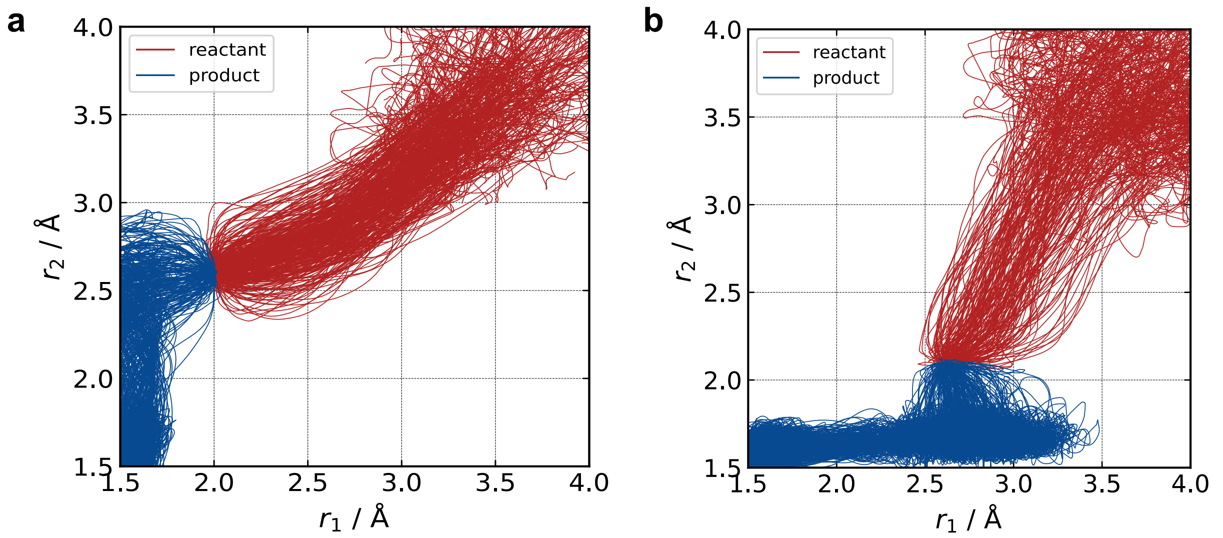

Figure S31: Distance between the two forming C-C bonds for downhill trajectories in (a) *endo* and (b) *exo* reaction in explicit water

Table S14: Summary of 500 downhill trajectories initiated from the TS structures in explicit methanol with PBC and NVT ensemble (300 K, time step = 0.5 fs)

	# reactant	# product	Average time gap	# product with time gap < 60 fs
endo	269 (54%)	231 (46%)	24.8 fs	218 (94% of # product)
exo	272 (54%)	228 (46%)	37.0 fs	189 (83% of # product)

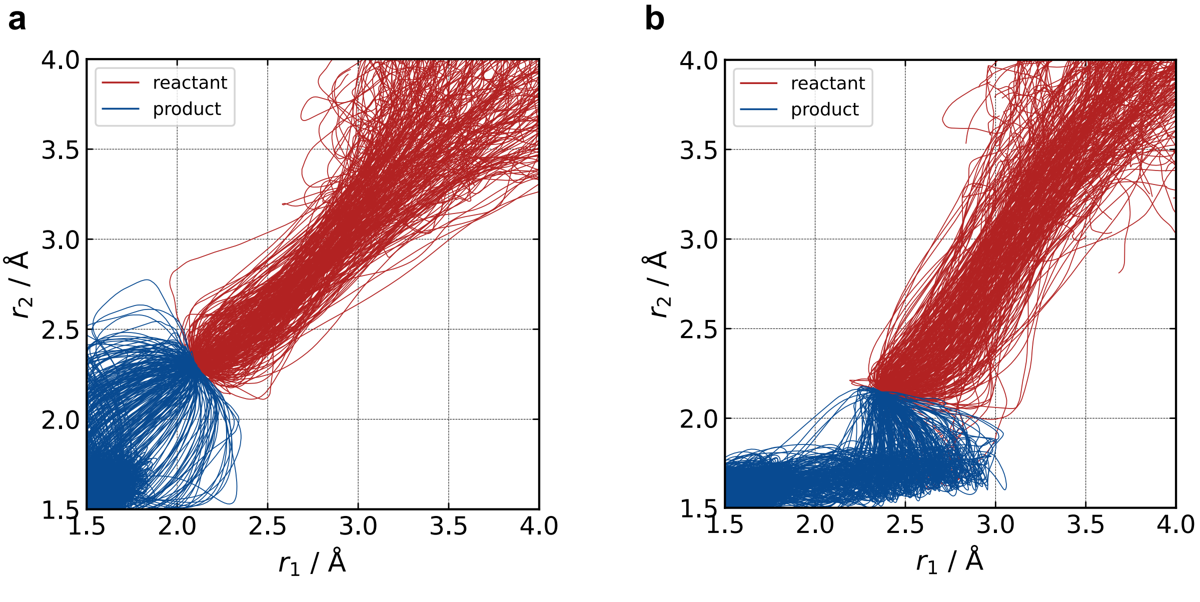

Figure S32: Distance between the two forming C-C bonds for downhill trajectories in (a) *endo* and (b) *exo* reaction in explicit methanol

S10.2 Uphill dynamics

The uphill trajectories were initialised from the reactants complex solvated in a PBC box with a box size of 21.5 Å, containing either 320 water molecules or 143 methanol molecules. A larger box was utilised here compared with the free energy calculations and downhill dynamics to ensure sufficient size to capture the bulk solvent performance. The uphill trajectories propagated with a harmonic potential added to r_1 and r_2 with a spring constant of 0.4 eV Å^{-2} to surmount the reaction barriers, utilising the NVT ensemble (at 300 K, a timestep of 0.5 fs and saving the trajectory every 1 fs). We performed 500 uphill trajectories in each solvent.

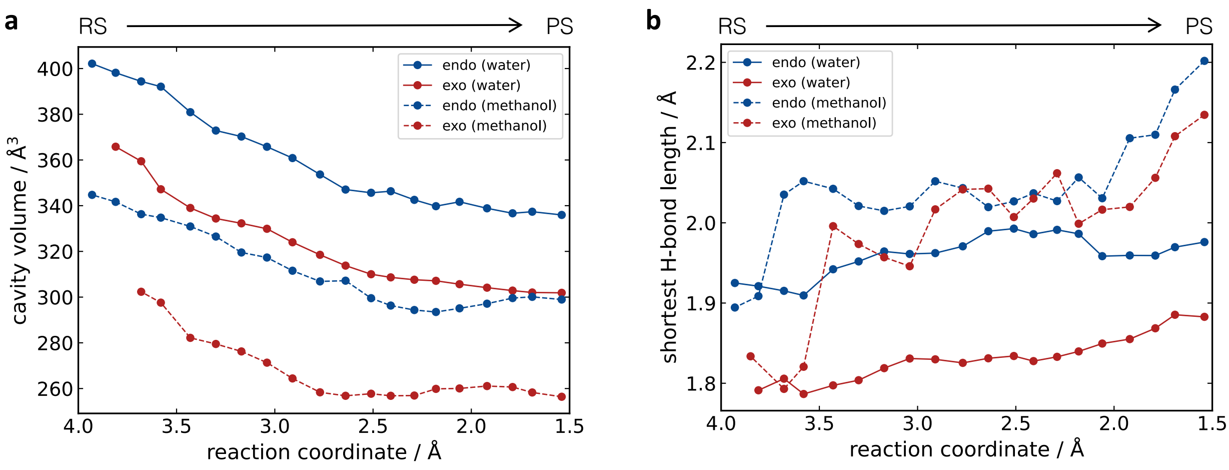

Figure S33: Uphill trajectories properties. (a) Cavity volumes (b) Shortest hydrogen bond length between carbonyl group in MVK and solvent molecules for *endo* and *exo* reactions in explicit water and methanol along the reaction coordinate. Both properties are represented as the mean values over the frames corresponding to the same reaction coordinate.

Two properties were monitored for each frame in the trajectories that form the product (where r_1 and r_2 are smaller than 1.6 Å) along a reaction coordinate of average bond length for r_1 and r_2: the cavity volume and the change in HBs. The cavity volume was determined by placing a set of grid boxes with a size of 0.2 Å in the frame and counting the number of boxes which were located at a distance from solvent molecules greater than the maximal atomic van der Waals radius (1.5 Å for water and 1.7 Å for methanol) and were not occupied by solvent molecules. The HB was considered to exist if the distance between the oxygen atom in the solvent and the oxygen atom of the carbonyl group in the reactive species was smaller than 3.5 Å and the angle formed by the O(carbonyl)–H(solvent)–O(solvent) was within the range of 120° to 180°. The HB of RS, TS and PS were characterised by the number of HB and shortest HB length. All properties were averaged over the frames corresponding to the same reaction coordinate.

As seen in Fig. S33, the cavity volume decreases going from reactants to TS (at approximately 2.5 Å reaction coordinate) and remains nearly constant afterwards. Since the reaction contains early

Table S15: Summary of the number of hydrogen bonds for reactant (RS), TS and product (PS) states for the *endo* and *exo* reactions in explicit water and methanol obtained from uphill trajectories

	RS	TS	PS
endo in water	2.0	1.9	1.6
exo in water	3.0	2.8	2.3
endo in methanol	1.0	1.0	1.0
exo in methanol	2.0	1.8	1.8

TS, i.e., TS resembling more RS than PS, the constant volume suggests that TS converges to the PS quickly within 200 fs without sufficient time for solvent reorganisation. A similar pattern is observed in the change in HBs. The shortest length of HB slightly increases for reactions in water; however, a more significant increase is seen for reactions in methanol, particularly when the reaction traverses the free energy barriers. Such an increase in methanol could be attributed to the weaker HB network in methanol compared to water and the larger size of methanol molecules, resulting in a larger time for reorganisation. It is worth noting that the uphill trajectories stop as soon as the product is formed. If the trajectories were allowed to propagate for a longer time after reaching the product state, we would likely observe a continuous decrease in cavity volumes and a more gradual increase in the shortest length of HBs as the solvent undergoes further re-organisation.

Regarding the number of hydrogen bonds along the reaction, we observed no significant change in any of the solvents as listed in Table S15. Particularly, there are no additional HBs observed in TS compared to RS.

We analyzed HB bond lengths and angles during the *endo* reaction in explicit water and methanol to gain insight into the details of HB interactions. Fig. S34 shows the distributions of HB bond length and angle for RS, TS and PS. Configurations for each state were selected from frames along the uphill trajectories using specific criteria: RS (with r_1 and r_2 larger than 3.00 Å), TS (with a reaction coordinate of 2.30 Å for water and 2.25 Å for methanol), and PS (with r_1 and r_2 smaller than 1.60 Å).

In both solvents, the RS exhibits narrower distributions of HB bond lengths and angles compared to the TS and PS. Methanol shows a larger increase in bond angle from RS to PS, with a change of 6.1°, compared to water, which has a change of 2.6°. It is important to note that although the mean value of the HB bond length for the TS in water is larger than that for the RS, there are some HBs with shorter bond lengths, indicating the presence of enhanced HBs at the TS. However, such enhanced HBs can be neglected in methanol.

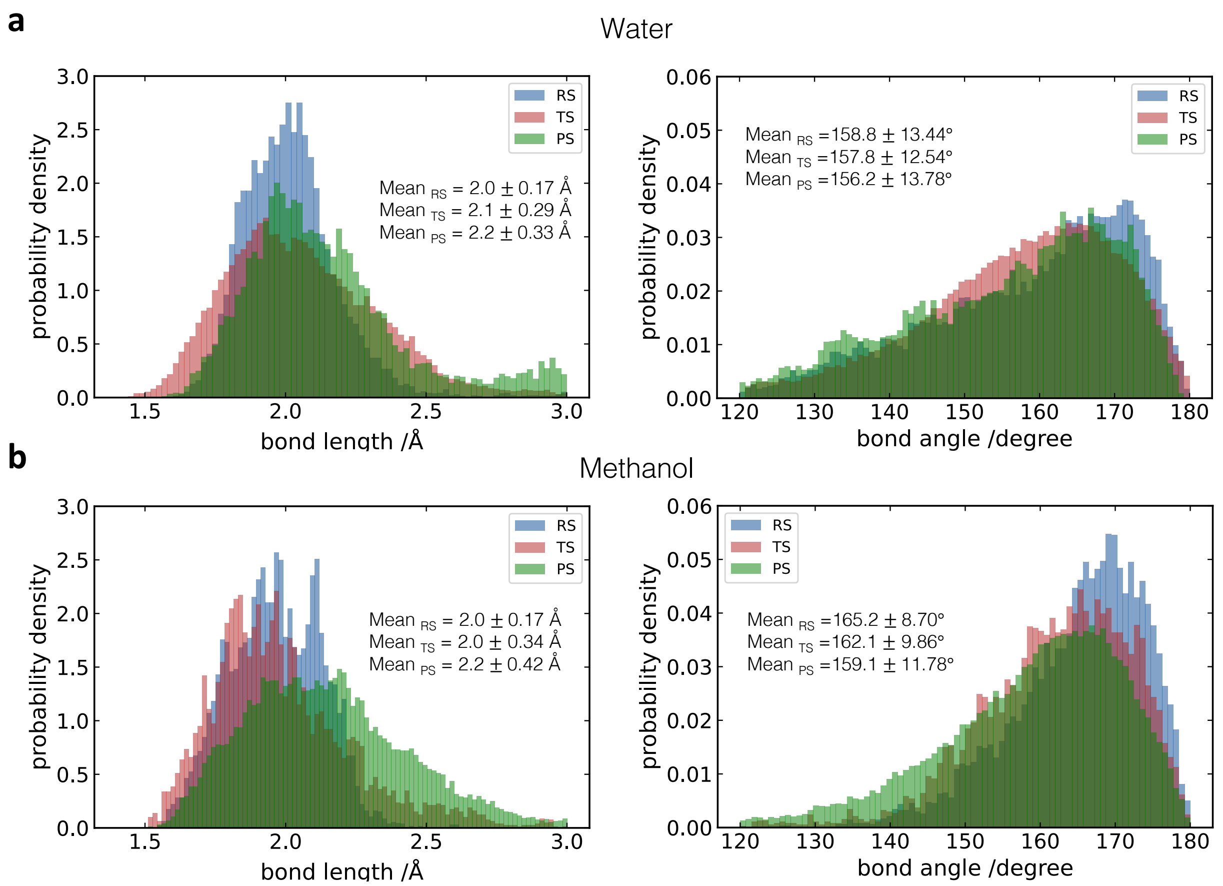

Figure S34: Hydrogen bonds properties (bond length and bond angle) distributions for the *endo* reaction in explicit (a) water and (b) methanol at RS, TS and PS. The configurations for each state are obtained from trajectories propagated by uphill dynamics

References

(1) Chen, Y.; Krämer, A.; Charron, N. E.; Husic, B. E.; Clementi, C.; Noé, F. *J. Chem. Phys.* **2021**, *155*, 084101.

(2) Noé, F.; Tkatchenko, A.; Müller, K.-R.; Clementi, C. *Annu. Rev. Phys. Chem* **2020**, *71*, 361–390.

(3) Gastegger, M.; Schütt, K. T.; Müller, K.-R. *Chem. Sci.* **2021**, *12*, 11473–11483.

(4) Shen, L.; Wu, J.; Yang, W. *J. Chem. Theory Comput.* **2016**, *12*, 4934–4946.

(5) Töpfer, K.; Käser, S.; Meuwly, M. *Phys. Chem. Chem. Phys.* **2022**, *24*, 13869–13882.

(6) Yang, M.; Bonati, L.; Polino, D.; Parrinello, M. *Catal. Today* **2022**, *387*, 143–149.

(7) Yang, X.; Zou, J.; Wang, Y.; Xue, Y.; Yang, S. *Eur. J. Chem.* **2019**, *25*, 8289–8303.

(8) Yang, Y.; Zhang, S.; Ranasinghe, K.; Isayev, O.; Roitberg, A. *ChemRxiv* **2023**, DOI: `10.26434/chemrxiv-2023-x82fz`.

(9) Yao, S.; Van, R.; Pan, X.; Park, J. H.; Mao, Y.; Pu, J.; Mei, Y.; Shao, Y. *RSC Adv.* **2023**, *13*, 4565–4577.

(10) Katzberger, P.; Riniker, S. *J. Chem. Phys.* **2023**, *158*, 204101.

(11) Böselt, L.; Thürlemann, M.; Riniker, S. *J. Chem. Theory Comput.* **2021**, *17*, 2641–2658.

(12) Rossi, K.; Jurásková, V.; Wischert, R.; Garel, L.; Corminbœuf, C.; Ceriotti, M. *J. Chem. Theory Comput.* **2020**, *16*, 5139–5149.

(13) Jurásková, V.; Célerse, F.; Laplaza, R.; Corminboeuf, C. *J. Chem. Phys.* **2022**, *156*, 154112.

(14) Zhou, B.; Zhou, Y.; Xie, D. *J. Chem. Theory Comput.* **2023**, *19*, 1157–1169.

(15) Devergne, T.; Magrino, T.; Pietrucci, F.; Saitta, A. M. *J. Chem. Theory Comput.* **2022**, *18*, 5410–5421.

(16) Young, T. A.; Johnston-Wood, T.; Deringer, V. L.; Duarte, F. *Chem. Sci.* **2021**, *12*, 10944–10955.

(17) Young, T. A.; Johnston-Wood, T.; Zhang, H.; Duarte, F. *Phys. Chem. Chem. Phys.* **2022**, *24*, 20820–20827.

(18) Adamo, C.; Barone, V. *J. Chem. Phys.* **1999**, *110*, 6158–6170.

(19) Grimme, S.; Ehrlich, S.; Goerigk, L. *J. Comput. Chem.* **2011**, *32*, 1456–1465.

(20) Weigend, F.; Ahlrichs, R. *Phys. Chem. Chem. Phys.* **2005**, *7*, 3297–3305.

(21) Singhal, A. *Bulletin of the IEEE Computer Society Technical Committee on Data Engineering* **2001**, *24*, 35–43.

(22) Krause, E. F., *Taxicab Geometry: Adventure in Non-Euclidean Geometry*; Dover Publications Inc.: 1988.

(23) Fink, R. F. *J. Chem. Phys.* **2010**, *133*, 174113.

(24) Linder, M.; Brinck, T. *Phys. Chem. Chem. Phys.* **2013**, *15*, 5108–5114.

(25) Neese, F. *Wiley Interdiscip. Rev. Comput. Mol. Sci.* **2018**, *8*, e1327.

(26) Grimme, S. *J. Chem. Phys.* **2006**, *124*, 034108.

(27) Zhao, Y.; Truhlar, D. *Theor. Chem. Acc.* **2008**, *120*, 215–241.

(28) Najibi, A.; Goerigk, L. *J. Chem. Theory Comput.* **2018**, *14*, 5725–5738.

(29) Lin, Y.-S.; Li, G.-D.; Mao, S.-P.; Chai, J.-D. *J. Chem. Theory Comput.* **2013**, *9*, 263–72.

(30) Chai, J.-D.; Head-Gordon, M. *J. Chem. Phys.* **2008**, *128*, 084106.

(31) Neese, F.; Wennmohs, F.; Hansen, A.; Becker, U. *Chem. Phys.* **2009**, *356*, 98–109.

(32) Schäfer, A.; Huber, C.; Ahlrichs, R. *J. Chem. Phys.* **1994**, *100*, 5829–5835.

(33) Weigend, F. *Phys. Chem. Chem. Phys.* **2006**, *8*, 1057–1065.

(34) Barone, V.; Cossi, M. *J. Phys. Chem. A* **1998**, *102*, 1995–2001.

(35) Acevedo, O.; Jorgensen, W. L. *J. Chem. Theory Comput.* **2007**, *3*, 1412–1419.

(36) Töpfer, K.; Käser, S.; Meuwly, M. *Phys. Chem. Chem. Phys.* **2022**, *24*, 13869–13882.

(37) Soto-Delgado, J.; Tapia, R. A.; Torras, J. *J. Chem. Theory Comput.* **2016**, *12*, 4735–4742.
